# Pep-3D-Search: a method for B-cell epitope prediction based on mimotope analysis

**DOI:** 10.1186/1471-2105-9-538

**Published:** 2008-12-16

**Authors:** Yan Xin Huang, Yong Li Bao, Shu Yan Guo, Yan Wang, Chun Guang Zhou, Yu Xin Li

**Affiliations:** 1 Institute of Genetics and Cytology, Northeast Normal University, Changchun 130024, PR China; 2 College of Computer Science and Technology, Jilin University, Changchun 130012, PR China; 3 Research Center of Agriculture and Medicine Gene Engineering of Ministry of Education, Northeast Normal University, Changchun 130024, PR China; 4 National Engineering Laboratory for Druggable Gene and Protein Screening, Northeast Normal University, Changchun 130024, PR China

## Abstract

**Background:**

The prediction of conformational B-cell epitopes is one of the most important goals in immunoinformatics. The solution to this problem, even if approximate, would help in designing experiments to precisely map the residues of interaction between an antigen and an antibody. Consequently, this area of research has received considerable attention from immunologists, structural biologists and computational biologists. Phage-displayed random peptide libraries are powerful tools used to obtain mimotopes that are selected by binding to a given monoclonal antibody (mAb) in a similar way to the native epitope. These mimotopes can be considered as functional epitope mimics. Mimotope analysis based methods can predict not only linear but also conformational epitopes and this has been the focus of much research in recent years. Though some algorithms based on mimotope analysis have been proposed, the precise localization of the interaction site mimicked by the mimotopes is still a challenging task.

**Results:**

In this study, we propose a method for B-cell epitope prediction based on mimotope analysis called Pep-3D-Search. Given the 3D structure of an antigen and a set of mimotopes (or a motif sequence derived from the set of mimotopes), Pep-3D-Search can be used in two modes: mimotope or motif. To evaluate the performance of Pep-3D-Search to predict epitopes from a set of mimotopes, 10 epitopes defined by crystallography were compared with the predicted results from a Pep-3D-Search: the average Matthews correlation oefficient (MCC), sensitivity and precision were 0.1758, 0.3642 and 0.6948. Compared with other available prediction algorithms, Pep-3D-Search showed comparable MCC, specificity and precision, and could provide novel, rational results. To verify the capability of Pep-3D-Search to align a motif sequence to a 3D structure for predicting epitopes, 6 test cases were used. The predictive performance of Pep-3D-Search was demonstrated to be superior to that of other similar programs. Furthermore, a set of test cases with different lengths of sequences was constructed to examine Pep-3D-Search's capability in searching sequences on a 3D structure. The experimental results demonstrated the excellent search capability of Pep-3D-Search, especially when the length of the query sequence becomes longer; the iteration numbers of Pep-3D-Search to precisely localize the target paths did not obviously increase. This means that Pep-3D-Search has the potential to quickly localize the epitope regions mimicked by longer mimotopes.

**Conclusion:**

Our Pep-3D-Search provides a powerful approach for localizing the surface region mimicked by the mimotopes. As a publicly available tool, Pep-3D-Search can be utilized and conveniently evaluated, and it can also be used to complement other existing tools. The data sets and open source code used to obtain the results in this paper are available on-line and as supplementary material. More detailed materials may be accessed at .

## Background

A B-cell epitope is defined as that part of antigen recognized by either a particular antibody molecule or a particular B-cell receptor of the immune system. It may be linear (continuous), i.e. a short contiguous stretch of amino acids, or conformational (discontinuous), consisting of sequence segments that are distantly scattered along the protein sequence and are brought together in spatial proximity when the protein is folded [1]. It has been estimated that more than ninety percent of B-cell epitopes are conformational [2,3]. The main purpose of B-cell epitope prediction is to provide the facilities for efficiently rational vaccine design [4]. Furthermore, synthetic peptides mimicking epitopes, as well as anti-peptide antibodies, have many applications in the diagnosis of human diseases [5,6]. Therefore B-cell epitope prediction is very important in medicine research.

Though B-cell epitopes can be directly identified using many biochemical or physical experiments, such as X-ray crystallography of antibody-antigen (Ab-Ag) complexes, these experiments are usually costly, time-consuming and are not always successful [7]. Computational methods to predict B-cell epitope are much more efficient and cost-effective. However they are mainly focused on the prediction of linear epitopes [8-14], because only few antigens are completely annotated with respect to their conformational epitopes, which makes it difficult to develop a conformational epitope prediction method. To the best of our knowledge, DiscoTope [15] and CEP [16] are the only two methods for conformational epitope prediction that are based on antigen structure information. Recently, researchers tested and evaluated existing epitope prediction methods on benchmark datasets, and concluded that the accuracies of these methods are not high enough to significantly reduce the experimental workload [17-19]. Combining experiments with computational methods can tremendously improve the accuracy of the epitope prediction at a modest cost in biological experiments. Therefore, it has attracted the attention of many researchers, especially in integrating computational methods with random peptide libraries. Several researchers have reported encouraging preliminary results using phage-display peptide libraries [20-29]. Mimotopes can be selected from phage-displayed random peptide libraries by affinity selection with monoclonal antibodies (mAb), so-called biopanning. The mAb affinity-selected mimotopes can be selected by their capacity of binding to the Ab directly against a given Antigen (Ag). Obviously, the mimotopes and Ag are both recognized by the same Ab paratope and thus mimotopes are expected to mimic natural epitopes. The purpose of the computational approach is to analyze the set of mimotopes and then to localize the mimicked region that is regarded as the epitope candidate. Thereafter, biological experiments, such as site-directed mutagenesis and deletion analysis, may be implemented for further validation.

Generally, a computational method has three steps to approach this goal: (i) the representation of the surface residues of the antigen; (ii) the search (or alignment) of the mimotopes (or motifs derived from the mimotopes) on the antigen surface; (iii) the output of the epitope candidates based on screening and clustering. Pizzi et al [20] were the first to combine computational methods with experimental results to assign epitopes. Recently, they published an improved method named MEPS [27]. In MEPS, the surface of antigen is represented by a collection of peptides below a certain length. The motifs that derived from the mimotopes are searched against this surface and alignment tools like BLAST can be directly used in the method. However, finding all given length simple paths (i.e. a sequence of neighboring residues) on a surface graph representing the exposed residues of the antigen is a NP-hard (Non-deterministic Polynomial-time hard) problem [29]. Subsequently, several computational algorithms were proposed, in which some new strategies were adopted [21-26,28,29]. For example, SiteLight [23] divides the antigen surface into overlapping patches and then aligns each mimotope with each patch based on the maximal bipartite matching algorithm. Mapitope [22,28] converts a set of mimotopes into overlapping residue pairs, then calculates them to rank the pairs' occurrences to obtain a set of major statistically significant pairs (SSP), and finally uses them to search the 3D structure of the antigen and links the SSP into clusters on the antigen surface. Lately, PepSurf [29], an epitope prediction program based on a color-coding algorithm [30], proposed to search all possible simple paths in the surface graph of an antigen and adopted a clustering strategy for epitope prediction. However, the running time of PepSurf depends exponentially on the length of a mimotope. Therefore, on their online server, each mimotope used must be less than or equal to 14 amino acids in length. Although epitopes and mimotopes are functionally equivalent, they seldom share a similar sequence. The mimicry is supposed to rely on similarities in physicochemical properties and similar spatial organization. Moreover, the binding site of an antibody is a surface, not just a continuous sequence, so the epitope prediction problem is outside the scope of classical string alignment algorithms. Searching all the surface residues on an antigen of interest for the mimotopes is problematical. Therefore, although numerous phage display library based algorithms have been proposed to characterize B-cell epitopes, the precise localization of the interaction site mimicked by the mimotopes on the antigen surface is still an open challenge [25,29].

In this research, we presented a method, Pep-3D-Search, based on mimotope analysis for B-cell epitope prediction. In Pep-3D-Search, a promising ACO (Ant Colony Optimization) algorithm was proposed to search matching paths on an antigen surface with respect to the query mimotopes or a motif. The ACO algorithm adopted a novel heuristic strategy that makes it powerful in dealing with longer mimotopes or motifs. Moreover, the P-value calculation algorithm and the DFS (Depth-First Search) algorithm, a graph search algorithm, were used to screen and cluster the result paths at the output stage. A group of test cases, which were all taken from published data, were applied to Pep-3D-Search for validation of its performance. The experimental results showed that the predictive performance of Pep-3D-Search was comparable to other epitope prediction algorithms, and some novel, rational results were provided.

## Implementation

### Algorithm flow

**Figure 1:**
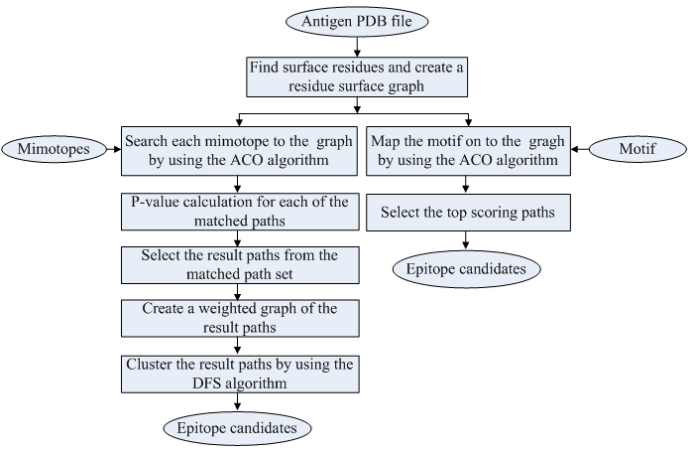
**An algorithmic flowchart of Pep-3D-Search**. Given the 3D structure of an antigen, Pep-3D-Search identifies all the surface residues and creates a surface graph. After that, it can be used in two modes: mimotope or motif. In mimotope mode, every mimotope received as an input is aligned to the antigen surface and the epitope candidates are obtained through screening and clustering of the matched paths. In motif mode, a motif received as an input is mapped on to the antigen surface. Subsequently, the top scoring paths are output directly as the epitope candidates.

The Pep-3D-Search algorithm flow is shown in Figure 1. Its input included a 3D structure of an antigen (a protein data bank (PDB) [31] file) and a set of mimotopes or a motif. Pep-3D-Search identified all exposed residues of the given antigen and created a surface graph of it. The algorithm can be employed in two modes. The first mode is the mimotope mode, which searched for matching paths on the antigen surface with each query mimotope by the ACO algorithm. All paths were scored to the corresponding mimotope according to an amino-acid substitution matrix. Putative candidate epitopes were then picked out by the P-value calculation algorithm and the DFS algorithm. The second mode is the motif mode, which directly mapped the motif onto the antigen surface using the ACO algorithm and took the top-scoring paths as epitope candidates.

### Graphical representation of the antigen surface

A B-cell epitope typically is a solvent accessible surface consisting of some 15–20 exposed residues derived from 2 to 3 discontinuous segments of the antigen [32]. Whether or not a residue is exposed can be determined by its solvent accessible surface area (SASA). In this study, the exposed residues in the study antigen were determined by three steps: (i) the total SASA of a residue composed of *N *atoms was calculated by: *SASA *= ∑_*N*_*A*_*i*_, where *A*_*i *_is the SASA of the *i*th atom and determined by the Surface Racer program 4.0 [33] with a probe sphere of radius 1.4 Å, corresponding to a water molecule; (ii) the relative solvent accessibility (RSA) of a residue was calculated as the *SASA *of the residue compared to the maximum exposed surface of the same residue type in an extended ALA-X-ALA tripeptide, where the maximum exposed surface of the residue X in the ALA-X-ALA tripeptide is that calculated by Ahmad al. [34]; (iii) A residue was determined as being exposed if the value of its RSA is greater than a predefined threshold (default = 5%). A surface graph representing the exposed residues, *G *= (*V*,*E*), was defined, where *V *is the vertex set consisting of all exposed residues, and *E *is the edge set, where any two vertices are connected by an edge if the Euclidian distance between the two vertices is not greater than a predefined threshold. In Pep-3D-Search, three methods were provided to calculate neighbor residue pairs on the antigen surface. Firstly the distance between the two residues was taken as the distance between the *C*_*α *_atoms of the two amino acids. Using *C*_*α *_atoms may better reflect the backbone positions. Secondly, the distance between the *C*_*β *_atoms was used, which may better reflect the side chain position (the *C*_*α *_atom was still used when it is a glycine because it does not have a *C*_*β *_atom). Thirdly, the minimum distances between all the heavy atoms of the two residues were used. In Pep-3D-Search, we used CA, CB and AHA to represent the three methods respectively and took CA as the default parameter with a distance threshold 7 Å.

### The ACO algorithm

ACO is a multi-agent heuristic algorithm used for combinatorial optimization. It was inspired by the capability of real ants to find the shortest path between their nest and a food source. The original ACO algorithm was introduced by Dorigo et al [35] for solving the traveling salesman problem (TSP). Since then, many researchers have extended the original algorithm, and have successfully applied their new algorithms to large scale TSP and other problems like the vehicle routing, scheduling, routing in Internet-like networks, and so on [36]. The successful application of ACO algorithms in the TSP inspired us to develop a new heuristic algorithm for solving the mimotope prediction problem. Our aim was to find a simple path on a surface graph that yielded the alignment to a mimotope or a motif with a maximal score. Similarly to the TSP, our problem was an ordering problem, i.e. the algorithm's aim was to put the different vertices in a certain order. However, several different aspects had to be considered: (i) our problem is a partial vertex permutation of a graph, in which the number of vertices in the permutation equals the residue number in the mimotope (or the motif); (ii) the edge of any two neighbor vertices must be the same length, and scoring a resulting path is only dependent on a vertex permutation, totally irrelevant to the path length; (iii) in a resulting path, some insertions/deletions may be permitted. Therefore, some new strategies were needed for solving our problem. The details of these strategies are described below.

### Definition of the pheromone trail and the heuristic information

The pheromone trail and the heuristic information are two important parameters in the ACO algorithm. Theoretically, the pheromone trail can give the artificial ants a global guide in their decision-making, whereas the heuristic information can guide these ants to explore better paths locally. The quality of an ACO application depends greatly on the definition of the meaning of the pheromone trail and the heuristic information [35]. According to the features of our problem, pheromone and the heuristic information for each edge on surface graph were defined as follows:

Let *τ*^(*k*)^(*i*, *j*) be the pheromone from vertex *i *to vertex *j *at the *k*th searching step in a solution, which encodes the favorability of visiting a certain vertex *j *after vertex *i*, where 1 ≤ *k *≤ *L*, and *L *is the number of vertices in a resulting path (i.e. the number of residues in the mimotope or motif). In our approach, *τ*^(*k*)^(*i*, *j*) was assigned an initial value at the start point and was updated after each iteration.

Let *η*^(*k*)^(*i*, *j*) be the heuristic information from vertex *i *to vertex *j *at the *k*th searching step in a solution, which encodes the preference of visiting a certain vertex *j *after vertex *i*, where 1 ≤ *k *≤ *L*, and *L *is the number of vertices in a resulting path. The value of *η*^(*k*)^(*i*, *j*) was assigned according to the input mimotope (or motif) and the amino-acid substitution matrix used (see Scoring amino acid similarities). For example, let the mimotope be "ANYNATRGTVSA", and a row of the amino-acid substitution matrix used is supposed to be: "A←A(2.14), K(0.44), I(0.39), G(0.25), V(0.07), D(-0.15), S(-0.22), N(-0.36), Q(-0.36), T(-0.4), F(-0.61), C(-0.61), E(-0.7), L(-0.73), M(-0.91), Y(-0.91), H(-1.15), P(-1.15), R(-1.67), W(-2.61)" which represents the scoring values of each amino-acid substitution for Alanine (A). It can be seen that the first, the fifth and twelfth amino acid in the mimotope are all alanine (A). In order to make the ants tend to find maximal alignment score in each step, for *k *= 1, 5 and 12, we will set *η*^(*k*)^(*i*, *j*) = 2.14 if the vertex *j *is a Alanine (A) and *i *is any neighbor vertex of *j*, and in the same way, *η*^(*k*)^(*i*, *j*) = 0.44, if the vertex *j *is a Lysine (K) and *i *is any neighbor vertex of *j*,..., finally, *η*^(*k*)^(*i*, *j*) = -2.61, if the vertex *j *is a Tryptophan (W) and *i *is any neighbor vertex of *j*. In this way, for all 1 ≤ *k *≤ 12 and each edge on the surface graph, *η*^(*k*)^(*i*, *j*) can be defined and it naturally represents the preference of an ant in vertex *i *for vertex *j *in each searching step.

In the case of a motif, let *Q *= (*q*_1_, *q*_2_,...,*q*_*L*_) be the motif, then *q*_*k *_(1 ≤ *k *≤ *L*) may be a set of amino acids (e.g. [STDE], see Epitope prediction based on motif mapping), a gap (-) or a character "X" which means it can be any amino acid. When *q*_*k *_is a set of amino acids (the set is named *S*), *η*^(*k*)^(*i*, *j*) will be set to be the maximal value in all the scoring values of vertex *i *substitution for vertex *j*, where the vertex *j *belongs to the set *S *and *i *is any neighbor vertex of *j*; When *q*_*k *_is a gap or a character "X", *η*(^*k*)^(*i*, *j*) will be set to be the average value of the substitution matrix, if *j *and *i *are a pair of neighbors.

### Scoring amino acid similarities

Algorithms for alignment of protein sequences typically measure similarity by using a substitution matrix with scores for all possible exchanges of one amino acid with another. The choice of the substitution matrix will directly influence the performance of the algorithms. However, the optimal substitution matrices used by the existing epitope prediction algorithms are generally not compatible with each other. Following comparison experiments, we chose the substitution matrix M_Blosum62 by Mayrose et al [29] as the default selection for the similar match mode. Moreover, we defined the substitution matrix STRICT as the default selection for the exact match mode, in which the scoring value of substitution between the same two amino acids is 1, whereas the scoring value of substitution between any two different amino-acids is 0. A simple path on the surface graph is a path in which all vertices are distinct. When an ant has no no-visited edge to connect to other vertices, it is allowed to jump to a no-edge-connected vertex if the distance between the two vertices is less than the double predefined distance threshold. In this situation, a gap can be left on its path. For each unmatched residue, a penalty was added.

According to the above analysis, two methods for scoring the similarity of amino acids are proposed. For mimotope analysis, the similarity score *h*(*q*_*i*_, *p*_*i*_) of amino acids *q*_*i *_and *p*_*i *_is calculated by Equation (1):

(1) $$h(q_{i},p_{i})=\{\begin{array}{ll} minimum+penalty & \text{if }q_{i}\text{ or }p_{i}\text{ is a gap}\\ s(q_{i},p_{i}) & \text{otherwise} \end{array}$$

Where *minimum *refers to the minimum value in the substitution matrix used; the values of *penalty *are set from 0 to -0.5 (default = -0.5); *s*(*q*_*i*_, *p*_*i*_) is the observed substitution score in the substitution matrix used.

In the case of motif analysis, let *Q *= (*q*_1_, *q*_2_,..., *q*_*L*_) be the motif and *P *= (*p*_1_, *p*_2_,..., *p*_*L*_) be the resulting path on the surface graph, then we calculate the similarity score *h*(*q*_*i*_, *p*_*i*_) (1 ≤ *i *≤ *L*) by Equation (2):

(2) $$h(q_{i},p_{i})=\{\begin{array}{ll} average & \text{if }q_{i}\text{ is X or}-(\text{a gap})\\ minimum+penalty & \text{if }q_{i}\text{ is an amino-acid and }p_{i}\text{ is a gap}\\ s(q_{i},p_{i}) & \text{if both }q_{i}\text{ and }p_{i}\text{ are amino-acids} \end{array}$$

Where *average *refers to the average value in the substitution matrix used; *minimum *denotes the minimum value in the substitution matrix used; the values of *penalty *is set from 0 to -0.5 (default = -0.5); *s*(*q*_*i*_, *p*_*i*_) is the observed substitution score in the substitution matrix used.

### Building a solution

The pheromone trail and the heuristic information defined above will now be used by the ants to find the best solutions. Suppose the number of residues in the mimotope is *L*. Every ant starts with a virtual original point named "O", which is permitted to connect to any vertex on the graph. Then an ant will randomly choose a vertex as its first vertex, and builds a solution going from a vertex to another connected vertex. The process will not stop until the ant has visited *L *vertices on the graph. At the *k*th searching step (1 ≤ *k *≤ *L*), the probability that an ant *A *in a vertex *i *will choose a vertex *j *as its next vertex is given by equation (3):

(3) $$P_{A}(i,j)=\{\begin{array}{ll} \frac{{\left[\tau^{\left(k\right)}(i,j)\right]}^{\alpha }{\left[\eta^{\left(k\right)}(i,j)\right]}^{\beta }}{\sum_{g\in J_{A}(i)}{\left[\tau^{\left(k\right)}(i,g)\right]}^{\alpha }{\left[\eta^{\left(k\right)}(i,g)\right]}^{\beta }} & \text{if }j\in J_{A}(i)\\ 0 & \text{otherwise} \end{array}$$

Where *τ*^(*k*)^(*i*, *j*) and *η*^(*k*)^(*i*, *j*) are the pheromone and the heuristic information between *i *and *j *at *k*th searching step, respectively. So the preference of an ant *A *in vertex *i *for vertex *j *is partly defined by the pheromone between *i *and *j*, and partly by the heuristic favorability of *j *after *i*. Parameters *α *and *β *define the relative importance of the pheromone information and the heuristic information (default *α *= *β *= 2). *J*_*A*_(*i*) is the set of vertices that connect to *i *and have not yet been visited by the ant *A *in vertex *i*.

### The fitness function

In order to guide the algorithm towards good solutions, a fitness function was defined to assess the quality of the solutions. Let *Q *= (*q*_1_, *q*_2_,..., *q*_*L*_) be a mimotope (or a motif) of length *L *and *P *= (*p*_1_, *p*_2_,...,*p*_*L*_) be a simple path on the surface graph obtained by an ant. Then, the alignment score between *Q *and *P *is defined as: $S(Q,P)=\sum_{i=1}^{L}h(q_{i},p_{i})$, where *h*(*q*_*i*_, *p*_*i*_) denotes the amino acid similarity score between *q*_*i *_and *p*_*i*_. Here, the average of the alignment score between *Q *and *P *is chosen to define the fitness of the solution *P*:

(4) F(P)=S(Q,P)L

### Updating the pheromone trail

After all the ants have completed one iteration, the pheromones were updated. Firstly, we defined the elite ant as follows: an ant was appointed as the elite ant only if the fitness value of the path obtained by the ant was greater than a threshold. Only the elite ants were permitted to leave the pheromones on its own path. The pheromones were updated according to equations (5) and (6).

(5) *τ*^(*k*)^(*i*, *j*) = (1 - *ρ*)*τ*^(*k*)^(*i*, *j*) + Δ*τ*(*i*, *j*)

(6) $$\Delta \tau (i,j)=\{\begin{array}{ll} F(P) & \text{if }(i,j)\in \text{path }P\text{ of the elite ant}\\ 0 & \text{otherwise} \end{array}$$

Equation (5) consists of two parts and *k *represents the *k*th searching step. The left part makes the pheromone on all edges decay. The speed of this decay is defined by the evaporation parameter *ρ *(0 <*ρ *< 1) (default *ρ *= 0.05). The right part increases the pheromones on all the edges visited by the elite ants. The amount of pheromone that the elite ant deposits on an edge is defined by the fitness value of the path created by the ant, as in equation (6). In this way, the increase of pheromone for an edge depends on the number of the elite ants that use this edge, and on the quality of the solutions found by those ants.

In order to enhance exploration of ants and overcome the premature convergence of the ACO algorithm, an adaptive strategy was employed to determine the threshold (which was used to select the elite ants): (i) initially, the threshold was set to 1; (ii) within 300 iterations, if the total number of the elite ants determined in each iteration was less than 5, then the new threshold was set to equal the original threshold minus 0.1; within 20 iterations, if the total number of the elite ants determined in each iteration was greater than 10, then the new threshold was set to equal the original threshold plus 0.1. In addition, according to Stützle and Hoos [37], we defined an upper and lower limit (*τ*_max _and *τ*_min_) for the pheromone values. Stützle and Hoos defined *τ*_max _and *τ*_min _algebraically based on the probability of constructing the best solution found when all the pheromone values have converged to either *τ*_max _or *τ*_min_. In our approach, the aim of the ACO algorithm was mainly to provide a set of good quality solutions, rather than a best solution. Therefore we defined *τ*_max _as being equal to the maximum value minus the minimum value in the amino-acid substitution matrix used, and *τ*_min _as zero.

### Output of epitope candidates

While running the ACO algorithm, all paths obtained by the elite ants were stored in a local database. How were putative epitope candidates produced from this set of paths? According to the different kinds of input sequences, i.e. a set of mimotopes or a motif, two different strategies were adopted. For the set of mimotopes, a clustering strategy was employed (described as next section); for the motif, the *n *highest scoring paths were chosen directly as the epitope candidates.

### P-value calculation for a path

Typically, a set of input mimotopes contains a number of amino-acid sequences with different lengths. In order to rationally assess the paths obtained with different mimotopes, we calculated the probability of randomly obtaining a path with a specific score, i.e. P-value of the path. According to the work by Mayrose et al [29], the distribution of the scores of random paths can be approximated using an extreme value distribution, whose parameters are fitted from the empirical distribution using the method of moments. To obtain rational empirical distribution of alignment scores, we generated a set of *m *(default *m *= 10^6^) random simple paths on the surface graph for every mimotope, and each random simple path was then aligned to the mimotope.

### Creating a weighted graph of the result paths

We then selected those paths whose P-values were less than or equal to 10^-3 ^as the result paths and created a weighted graph of the result paths *G *= (*V*, *E*), where *V *is the vertex set consisting of all the result paths, and *E *is the edge set, where any two vertices are connected by an edge if they share at least one residue. In addition, the weight of each vertex in *G *was defined as the P-value of the path.

### Clustering the result paths based on DFS algorithm

The weighted graph defined above was generally unconnected. Each connection component in the graph, which may consist of several connected paths, can be regarded as a potential epitope candidate. Here, the DFS algorithm [30] was employed to compute all the connection components of the weighted graph. According to Mayrose et al [29], the surface accessible areas of 95% of all available epitopes in the PDB are not greater than 2000 Å^2^. Moreover, a native epitope is generally less than 40 residues. Therefore, if the surface accessible area of a connection component was greater than 2000 Å^2 ^or the number of residues in the connection component was greater than 40, this connection component was reduced in size. By iteratively removing a path, the size was cut until the remaining part met the conditions. In each such iteration, the algorithm chose a path for removal such that the remaining connection components kept the maximum score. The score of a connection component was defined as the sum of -log (P-value) of the paths within it. As a consequence, *n *maximum score connection components were output as the *n *epitope candidates (default *n *= 3).

## Results

### Epitope prediction based on mimotope analysis

In order to assess the predictive performance of Pep-3D-Search, we applied it to ten test cases (see Table 1), which were all taken from other similar published data. These test cases fulfilled the following requirements: (i) a set of mimotopes were derived by screening an antibody in a biopanning experiment; (ii) a 3D structure of the antibody-antigen complex was available; (iii) the native epitope of each test case had been crystallographically defined. Due to the similar policy of fully scanning the mimotopes (or neighbor amino acid pair (AAP) derived from the mimotopes in Mapitope [22,28]) versus the 3D structure of the antigen, we mainly compared the results from Pep-3D-Search with those from PepSurf [29] and Mapitope.

**Table 1:** The test cases used for Assessment of Pep-3D-Search's performance in mimotope anlysis.

**PDB ID**	**Antibody**	**Antigen**	**References**	Library size*
**Antibody-antigen test cases**				
1jrh	mAb A6	IFNgammaR	Lang S et al.(2000)	59 × 5
1bj1	rhuMAb VEGF	vascular endothelial growth factor	ChenY et al. (1999)	36 × 6, 3 × 5, 2 × 4
1g9m	mAb 17b	gp120	Enshell-Seijffers D et al. (2003)	10 × 14,1 × 12
1e6j	mAb 13b5	p24	Enshell-Seijffers D et al. (2003)	14 × 14, 2 × 7
1n8z	Herceptin Fab	Her-2	Riemer AB et al. (2004)	5 × 12
1iqd	mAb Bo2C11	Coagulation factor VIII	Villard S et al. (2003)	27 × 12
1yy9	Cetuximab Fab	Epidermal Growth Factor Receptor	Riemer AB et al. (2005)	4 × 10
2adf	82D6A3 IgG	Von Willebrand factor	Vanhoorelbeke K et al. (2003)	2 × 15, 3 × 6
**Protein-protein test cases**				
1avz	Fyn SH3 domain	Nef	Rickles RJ et al. (1994)	8 × 10, 10 × 12
1hx1	Bovine Hsc70	Bag chaperone regulator	Takenaka IM et al. (1995)	8 × 15

*Number of sequences × sequence length.

### Epitope prediction using antibody-antigen test cases

The first test group (antibody-antigen test cases in Table 1) contained eight test cases from Mapitope, PepSurf and Mimox [26]. The first test case (labeled 1jrh in Table 1) contains 59 mimotopes of 5 residues in length. Lang et al [38] further analyzed the detailed interactions between the mAb A6 and the interferon gamma receptor (IFNgR) by selecting 59 fragments of the IFNgR mutants with high affinity for the mAb A6 by phage display. These fragments can thus be regarded as mimotopes of the IFNgR and the crystal structure of the mAb A6-IFNgR complex has been resolved (PDB id: 1jrh). In the second test case (labeled 1bj1 in Table 1), mimotopes were obtained by a similar experiment to the first case, but here the Fab fragment of a humanized neutralizing antibody (also known as rhuMAb VEGF) was mutated and selected for binding to the vascular endothelial growth factor (VEGF) by phage display [39]. The structure of the rhuMAb-VEGF complex has been deposited in the PDB (PDB id: 1bj1). In test cases three to eight, the six sets of mimotopes were obtained by screening phage display libraries with the 17b [22], 13b5 [22], Herceptin [40], Bo2C11 [41], Cetuximab Fab [42] and 82D6A3 IgG [43] antibodies respectively (see Table 1), and their corresponding Ab-Ag complex structures have been resolved (PDB id: 1g9m, 1e6j, 1n8z, 1iqd, 1yy9 and 2adf). In addition, the native epitope for each test case (1–8) is present in the CED database [44]. We analyzed the mimotopes in the test cases with our Pep-3D-Search, PepSurf and Mapitope, respectively. The results predicted by the three algorithms and evaluation in terms of the Matthews correlation coefficient (MCC) [45], sensitivity and precision are shown in Table 2. The results in Table 2 show that our Pep-3D-Search successfully predicted all the mimotopes in all eight test cases. Especially, for the test cases 1bj1, 1n8z and 1yy9, the MCC, sensitivity and precision values of Pep-3D-Search were considerably superior to those of PepSurf and Mapitope. For the test case 1iqd, PepSurf yielded the best performance (MCC: 0.1272; sensitivity: 0.2581; precision: 0.5); though Mapitope achieved the highest precision (0.9375), it gave the lowest MCC (-0.3502) and sensitivity (0.1415); Pep-3D-Search yielded inferior prediction (MCC: 0.0356; sensitivity: 0.1277; precision: 0.375) with default parameters, whereas it obtained better prediction by using distance parameter CB with threshold 6.5 (MCC: 0.1604; sensitivity: 0.2326; precision: 0.625, see Table 3). Furthermore, for the test cases 1jrh, 1g9m, 1e6j and 2adf, Pep-3D-Search and PepSurf gave better predictions, while Mapitope failed in the test cases 1e6j and 2adf.

**Table 2:** Evaluation and comparison of the performances of Pep-3D-Search.

**PDB ID**	1jrh	1bj1	1g9m	1e6j	1n8z	1iqd	1yy9	2adf	1avz	1hx1	**Average**
**CED ID**	CE0179	CE0175	CE0058	CE0170	CE0096	CE0176	CE0199	CE0154	--	--	
**Epitope size**	21	19	15	11	20	16	15	15	16	24	
**Antigen size**	94	93	304	209	580	155	612	188	102	111	
**Pep-3D-Search**											
**TP/PE**	19/40	7/13*	10/39	11/36	20/35*	6/47	10/25	12/36	10/39	13/39	
**MCC**	0.3902	0.1442	0.1394	0.2285	0.1856	0.0356	0.1030	0.2153	0.1643	0.152	**0.1758**
**Sensitivity**	0.475	0.5833	0.2564	0.3056	0.5714	0.1277	0.4	0.3333	0.2564	0.3333	0.3642
**Precision**	0.9048	0.3684	0.6667	1.0	1.0	0.375	0.6667	0.8	0.625	0.5417	**0.6948**
**PepSurf**											
**TP/PE**	19/28	2/17	9/31	10/30	6/11	8/31	1/8*	10/18	14/25	12/25	
**MCC**	0.4134	-0.0537	0.1257	0.2056	0.0596	0.1272	0.0067	0.1832	0.3348	0.1863	0.1589
**Sensitivity**	0.6786	0.1176	0.2903	0.3333	0.5455	0.2581	0.125	0.5556	0.56	0.48	**0.3944**
**Precision**	0.9048	0.1053	0.6	0.9091	0.3	0.5	0.0476	0.6667	0.875	0.5	0.5409
**Mapitope**											
**TP/PE**	19/22	2/18	13/33	1/6	9/13	15/106	3/23*	0/10	6/9*	5/21	
**MCC**	0.4224	-0.062	0.1899	0.0154	0.0909	0.2401	0.0209	-0.0173	0.1387	0.0135	0.1053
**Sensitivity**	0.8636	0.1111	0.3939	0.1667	0.6923	0.1415	0.1304	0.0	0.6667	0.2381	0.3404
**Precision**	0.9048	0.1053	0.8667	0.0909	0.45	0.9375	0.1429	0.0	0.375	0.2083	0.4081

TP: number of true positives; PE: number of residues in the predicted epitope; TN: number of true negatives; FP: number of false positives; FN: number of false negatives; Matthews correlation coefficient $(\text{MCC})=\frac{\left(TP\cdot TN)-(FP\cdot FN\right)}{\sqrt{\left(TP+FP)(TP+TN)(TN+FP)(TN+FN\right)}}$; $\text{Sensitivity }(\text{Se})=\frac{TP}{TP+FN}$; $\text{Precision }(\text{Pr})=\frac{TP}{TP+FP}$.

*For the cases, the best prediction was found in the second-ranked candidate.

**Table 3:** Comparison of the predictive performance of Pep-3D-Search with different distance parameters (CB).

**PDB ID**	1jrh	1bj1	1g9m	1e6j	1n8z	1iqd	1yy9	2adf	1avz	1hx1	**Average**
**CB (distance threshold = 6.5)**											
**TP/PE**	5/5	0/0	11/43	11/38	15/30*	10/43	6/28	8/29	8/27	2/27	
**MCC**	0.1119	0.0	0.155	0.2285	0.1379	0.1604	0.059	0.1316	0.1380	-0.1271	0.0995
**Sensitivity**	1.0	0.0	0.2558	0.2895	0.5	0.2326	0.2143	0.2759	0.2963	0.0741	0.3139
**Precision**	0.2381	0.0	0.7333	1.0	0.75	0.625	0.4	0.5333	0.5	0.0833	0.4863
**CB (distance threshold = 7)**											
**TP/PE**	13/18	6/9*	14/46	8/28	14/31*	9/46	9/29*	14/31	9/41	11/38	
**MCC**	0.2747	0.1283	0.2048	0.1593	0.1282	0.127	0.0917	0.2604	0.1139	0.0939	0.1582
**Sensitivity**	0.7222	0.6667	0.3043	0.2857	0.4516	0.1957	0.3103	0.4516	0.2195	0.2895	0.3897
**Precision**	0.619	0.3158	0.9333	0.7273	0.7	0.5625	0.6	0.9333	0.5625	0.4583	0.6412
**CB (distance threshold = 7.5)**											
**TP/PE**	19/41	12/27	10/40	9/35	14/33**	2/45	8/28*	10/45	8/29	9/29	
**MCC**	0.3879	0.2349	0.1391	0.1806	0.1279	-0.0837	0.0809	0.1626	0.13	0.084	0.1444
**Sensitivity**	0.4634	0.4444	0.25	0.2571	0.4242	0.0444	0.2857	0.2222	0.2759	0.3103	0.2978
**Precision**	0.9048	0.6316	0.6667	0.8182	0.7	0.125	0.5333	0.6667	0.5	0.375	0.5921
**CB (distance threshold = 8)**											
**TP/PE**	19/38	15/30	10/37	4/34*	18/37	4/42	7/26	10/18*	6/27	11/32	
**MCC**	0.3947	0.3222	0.1401	0.0571	0.1664	-0.0101	0.0703	0.1832	0.0665	0.1271	0.1518
**Sensitivity**	0.5	0.5	0.2703	0.1176	0.4865	0.0952	0.2692	0.5556	0.2222	0.3438	0.3361
**Precision**	0.9048	0.7895	0.6667	0.3636	0.9	0.25	0.4667	0.6667	0.375	0.4583	0.5841

TP: number of true positives; PE: number of residues in the predicted epitope; TN: number of true negatives; FP: number of false positives; FN: number of false negatives; Matthews correlation coefficient $(\text{MCC})=\frac{\left(TP\cdot TN)-(FP\cdot FN\right)}{\sqrt{\left(TP+FP)(TP+TN)(TN+FP)(TN+FN\right)}}$; $\text{Sensitivity }(\text{Se})=\frac{TP}{TP+FN}$; $\text{Precision }(\text{Pr})=\frac{TP}{TP+FP}$.

*For the cases, the best prediction was found in the second-ranked candidate.

**For the cases, the best prediction was found in the third-ranked candidate.

### Using Pep-3D-Search for the prediction of protein-protein interacting sites

In order to compare Pep-3D-Search with previously published algorithms, we applied it to detect the interface residues of the interacting proteins for the two test cases, 1avz and 1hx1 (protein-protein test cases in Table 1), which were taken from PepSurf. Rickles et al [46] used the Fyn-SH3 domain to select a semi-combinatorial random peptide library and obtained 18 affinity-selected peptides. The co-crystal of Fyn-SH3 domain with its interacting protein Nef and Fyn-SH2 domain is now available (PDB id: 1avz). The second test case was taken from the work by Takenaka et al. [47]. They screened a random phage library against the 70 kDa heat shock cognate (Hsc70) protein and obtained a set of peptides that bind Hsc70. The structure of Hsc70 with its interacting protein Bag chaperone regulator has been deposited in the PDB (PDB id: 1hx1). For each of the above test cases, the prediction was compared to the 'true' protein-protein interacting site that was inferred using the 'Contact Map Analysis' server [48].

From Table 2, it can be seen that both Pep-3D-Search and PepSurf obtained better results than Mapitope. Especially, for the test case 1hx1, the results showed a complementarity between Pep-3D-Search and PepSurf: the 24 contacting residues of protein Hsc70 and Bag chaperone regulator inferred by Contact Map Analysis server were **R205 KA (208–209) IE (211–212) MK (215–216) LE (218–219) IDTLIL (221–226) R234 RK (237–238) VK (241–242) Q245 L248 D252 E255**; the 39 contacting residues predicted by Pep-3D-Search were GNS (150–152) E155 V157 K161 H164 K167 K171 AD (173–174) L200 K202 D204 **R205 **R206 **KA (208–209) I211 M215 L218 **FKD (230–232) **R234 **LK (235–236) **RK (237–238) **G239 **VK (241–242) **K243 **Q245 **AF (246–247) **L248 **AE (249–250); the 25 contacting residues suggested by PepSurf were K161 KHL (163–165) KS (167–168) E182 GI (185–186) D204 **R205 **R206 **KA (208–209) I211 MK (215–216) **I217 **LE (218–219) **E220 **DT (222–223) L248 E255**. From the above results, it is evident that in the predicted results of Pep-3D-Search, six epitope residues **R234**, **R237**,**K238**, **V241**, **K242 **and**Q245 **were missed by PepSurf, while in the predicted results of PepSurf, five epitope residues **K216**,**E219**, **D222**,**T223 **and **E255 **were missed by Pep-3D-Search.

**Figure 2:**
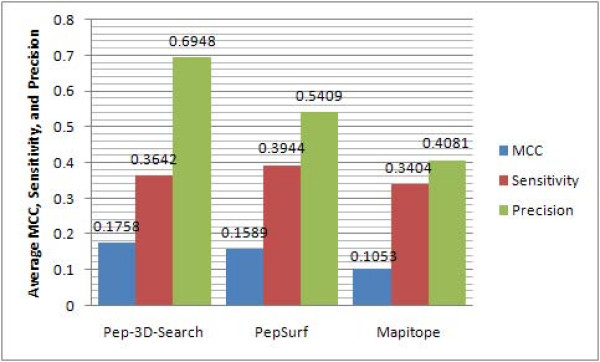
**Overall performance evaluation of Pep-3D-Search using average MCC, sensitivity and precision values**. From Figure 2, it can be seen that Pep-3D-Search obtained the best average MCC, precision values and second-best average sensitivity value; PepSurf obtained the best average sensitivity value and second-best average MCC and precision values; Mapitope gave inferior results in comparison with the above two methods.

**Figure 3:**
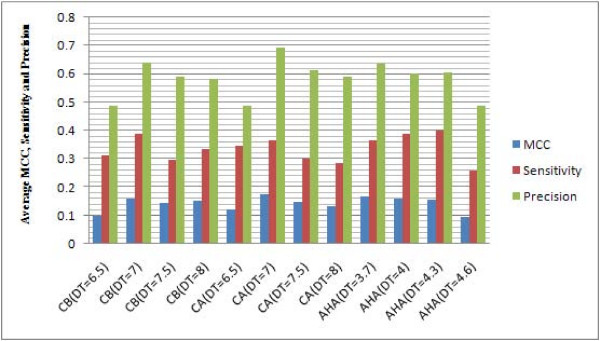
**Overall performance analysis of Pep-3D-Search with different distance parameters CB, CA and AHA**. From Figure 3, it can be seen that with parameter CA (DT (distance threshold) = 7), Pep-3D-Search obtained the best average MCC value (0.1758), precision value (0.6948), and the better average sensitivity (0.3642). In Pep-3D-Search the parameter CA with distance threshold 7 is set as the default.

The overall performance of each method was measured by average MCC, sensitivity and precision values. Compared with PepSurf and Mapitope, Pep-3D-Search achieved the best average MCC, precision values and second-best average sensitivity value (average MCC, sensitivity and precision values of predicted results by Pep-3D-Search were 0.1758, 0.3642, 0.6948; PepSurf were 0.1589, 0.3944 and 0.5409; Mapitope were 0.1053, 0.3404 and 0.4081, see Figure 2). In addition, Pep-3D-Search provides three parameters to calculate neighbor residue pairs on antigen surface, which are CB, CA and AHA. The experimental results that examined Pep-3D-Search's performance with different parameters are listed in Table 3 to 5. The overall performance analyses in terms of average MCC, sensitivity and precision values are shown in Figure 3. Generally, Pep-3D-Search obtained better results by using the parameter CA (distance threshold = 7) than by the other parameters. Subsequently the parameter CA with distance threshold 7 was set as the default.

**Table 4:** Comparison of the predictive performance of Pep-3D-Search with different distance parameters (CA).

**PDB ID**	1jrh	1bj1	1g9m	1e6j	1n8z	1iqd	1yy9	2adf	1avz	1hx1	**Average**
**CA (distance threshold = 6.5)**											
**TP/PE**	5/5	2/10	13/42	10/43	18/36*	7/31	2/10*	0/20	12/31	13/31	
**MCC**	0.1119	-0.0014	0.1887	0.2033	0.1664	0.1015	0.019	-0.0367	0.262	0.1889	0.1204
**Sensitivity**	1.0	0.2	0.3095	0.2326	0.5	0.2258	0.2	0.0	0.3871	0.4194	0.3474
**Precision**	0.2381	0.1053	0.8667	0.9091	0.9	0.4375	0.1333	0.0	0.75	0.5417	0.4882
**CA (distance threshold = 7)**											
**TP/PE**	19/40	7/13*	10/39	11/36	20/35*	6/47	10/25	12/36	10/39	13/39	
**MCC**	0.3902	0.1442	0.1394	0.2285	0.1856	0.0356	0.1030	0.2153	0.1643	0.152	**0.1758**
**Sensitivity**	0.475	0.5833	0.2564	0.3056	0.5714	0.1277	0.4	0.3333	0.2564	0.3333	**0.3642**
**Precision**	0.9048	0.3684	0.6667	1.0	1.0	0.375	0.6667	0.8	0.625	0.5417	**0.6948**
**CA (distance threshold = 7.5)**											
**TP/PE**	19/38	12/27*	10/45	9/33	18/40	0/36	7/25	12/36	9/37	9/36	
**MCC**	0.3947	0.2349	0.1374	0.1812	0.1662	-0.0895	0.0704	0.2153	0.1332	0.0411	0.1485
**Sensitivity**	0.5	0.4444	0.2222	0.2727	0.45	0.0	0.28	0.3333	0.2432	0.25	0.2996
**Precision**	0.9048	0.6316	0.6667	0.8182	0.9	0.0	0.4667	0.8	0.5625	0.375	0.6126
**CA (distance threshold = 8)**											
**TP/PE**	20/39	12/28*	10/40	10/35	17/36	0/37	5/26	13/35	8/39	5/32	
**MCC**	0.4248	0.2309	0.1391	0.2047	0.1565	-0.1144	0.0484	0.2378	0.0822	-0.065	0.1345
**Sensitivity**	0.5128	0.4286	0.25	0.2857	0.4722	0.0	0.1923	0.3714	0.2051	0.1563	0.2874
**Precision**	0.9524	0.6316	0.6667	0.9091	0.85	0.0	0.3333	0.8667	0.5	0.2083	0.5918

TP: number of true positives; PE: number of residues in the predicted epitope; TN: number of true negatives; FP: number of false positives; FN: number of false negatives; Matthews correlation coefficient $(\text{MCC})=\frac{\left(TP\cdot TN)-(FP\cdot FN\right)}{\sqrt{\left(TP+FP)(TP+TN)(TN+FP)(TN+FN\right)}}$; $\text{Sensitivity }(\text{Se})=\frac{TP}{TP+FN}$; $\text{Precision }(\text{Pr})=\frac{TP}{TP+FP}$.

*For the cases, the best prediction was found in the second-ranked candidate.

**Table 5:** Comparison of the predictive performance of Pep-3D-Search with different distance parameters (AHA).

**PDB ID**	1jrh	1bj1	1g9m	1e6j	1n8z	1iqds	1yy9	2adf	1avz	1hx1	**Average**
**AHA (distance threshold = 3.7)**											
**TP/PE**	17/20	7/16	12/44	11/34	17/36*	6/32	6/25*	9/33	13/36	8/32	
**MCC**	0.375	0.1243	0.1716	0.2286	0.1565	0.0733	0.0595	0.1502	0.2855	0.0351	0.1659
**Sensitivity**	0.85	0.4375	0.2727	0.3235	0.4722	0.1875	0.24	0.2727	0.3611	0.25	0.3667
**Precision**	0.8095	0.3684	0.8	1.0	0.85	0.375	0.4	0.6	0.8125	0.3333	0.6349
**AHA (distance threshold = 4)**											
**TP/PE**	16/20	7/10	13/42	8/30	15/30*	5/39	6/23	10/37	12/35	10/34	
**MCC**	0.3491	0.1528	0.1887	0.1584	0.1379	0.0283	0.0598	0.1695	0.2525	0.0858	0.1583
**Sensitivity**	0.8	0.7	0.3095	0.2667	0.5	0.1282	0.2609	0.2703	0.3429	0.2941	0.3873
**Precision**	0.7619	0.3684	0.8667	0.7273	0.75	0.3125	0.4	0.6667	0.75	0.4167	0.6021
**AHA (distance threshold = 4.3)**											
**TP/PE**	16/18	8/11	14/42	8/36	17/30*	9/37	4/16*	11/37	10/33	4/26	
**MCC**	0.3537	0.1773	0.2052	0.1558	0.1571	0.1431	0.0394	0.1923	0.1869	-0.0514	0.1559
**Sensitivity**	0.8889	0.7273	0.3333	0.2222	0.5667	0.2432	0.25	0.2973	0.303	0.1538	0.3986
**Precision**	0.7619	0.4211	0.9333	0.7273	0.85	0.5625	0.2667	0.7333	0.625	0.1667	0.6048
**AHA (distance threshold = 4.6)**											
**TP/PE**	19/36	9/24	9/44	4/41	18/38	0/34	8/25*	8/36*	5/37	6/27	
**MCC**	0.3989	0.1483	0.1206	0.0495	0.1663	-0.1023	0.0813	0.1241	-0.0357	0.0051	0.0956
**Sensitivity**	0.5278	0.375	0.2045	0.0976	0.4737	0.0	0.32	0.2222	0.1351	0.2222	0.2578
**Precision**	0.9048	0.4737	0.6	0.3636	0.9	0.0	0.5333	0.5333	0.3125	0.25	0.4871

TP: number of true positives; PE: number of residues in the predicted epitope; TN: number of true negatives; FP: number of false positives; FN: number of false negatives; Matthews correlation coefficient $(\text{MCC})=\frac{\left(TP\cdot TN)-(FP\cdot FN\right)}{\sqrt{\left(TP+FP)(TP+TN)(TN+FP)(TN+FN\right)}}$; $\text{Sensitivity }(\text{Se})=\frac{TP}{TP+FN}$; $\text{Precision }(\text{Pr})=\frac{TP}{TP+FP}$.

*For the cases, the best prediction was found in the second-ranked candidate.

### Epitope prediction based on motif mapping

Pep-3D-Search also provides the selection of predicting epitope based on motif mapping. The motif sequence can be derived from the set of mimotopes by using multiple sequence alignment tools such as ClustalW [49] or directly using the Mimox web service, and it is thus supposed to contain important residues for interaction of the Ab and the Ag. After mapping the motif sequence on to the antigen surface, Pep-3D-Search obtained a set of matched paths and those top-scoring paths were selected as the epitope candidates. In order to assess the performance of Pep-3D-Search, six test cases were applied and the results are listed in Table 6 and Supplementary Table S1 to S5 [see Additional file 1]. Here, we describe one experiment of the test case 1e6j (Table 6) in detail. The test case 1e6j is taken from Mapitope and Mimox. Enshell-Seijffers et al [22] used the mAb 13B5 (recognizing HIV-1 capsid protein p24) to select a phage displayed random peptide library and obtained a set of 16 mimotopes. The structure of p24 with 13B5 has been resolved [PDB: 1e6j], and the 13B5 epitope, which is composed of ALGPAATEE (204–210, 212, 213) TA (216–217), has been recorded in the CED database as CE0170. Using Mapitope, Enshell-Seijffers et al suggested that 13B5 epitope residues might consist of E187 D197 **A204 GPAA (206–209) EE (212–213) A217**, in which the epitope residues are marked in bold. It should be noted that when all parameters were set to default, Mapitope predicted candidate residues A194 N195 P196 D197 C198 **A217 **(i.e. among the six predicted residues, only one was epitope residue). Furthermore, Huang J et al [26] derived a motif sequence, [DE] V [FM] GPL [STDE] TX-X [DE], from the 16 mimotopes using Mimox. Mimox has no ability to directly analyze the motif sequence of this type, therefore they derived three fragments, GPL, ET and EE, from the motif by manual parsing. Using the three fragments as the motif sequences respectively, they predicted the 13B5 epitope using MIMOX. For the fragment GPL, the top two candidates given by MIMOX were **G206 P207 L205 **and G106 P49 L52; for the fragments ET, the top three candidates were **E212 T216**, **E213 T216 **and **E212 T210**; for the fragments EE, the top three candidates were E28 E29, E29 E28 and **E212 E213**. Using Pep-3D-Search we directly mapped the motif sequence, [DE]V [FM]GPL [STDE]TX-X [DE], on to the antigen surface of p24 to predict the 13B5 epitope. Under the similar match mode (i.e. using substitution matrix M_Blosum62, see Scoring amino acid similarities) and parameter AHA (distance threshold = 4), the top ten predicted candidates by Pep-3D-Search are listed in Table 6. From Table 6, we can see that the ten candidates all successfully localized in the epitope region. Especially, the eighth-ranked candidate gave the best results: D197 I201 **L205 G206 P207 A209 E213 T210 **M214 **A217 T216 E212**. Taking the top ten candidates together, we obtained a total of 25 residues suggested by Pep-3D-Search, which overlap 10 of the 11 epitope residues in the 13B5. The other five experiments for assessing the performance of Pep-3D-Search are similar to the procedure mentioned above, and their results are listed in Supplementary Tables S1 to S5 [see Additional file 1]. These experiments show that Pep-3D-Search is effective and efficient in predicting epitopes in motif mode.

**Table 6:** Epitope prediction of the test case 1e6j (chain: P) based on motif mapping : motif sequence taken from Mimox is [DE]V [FM]GPL [STDE]TX-X [DE]; native epitope recorded in CED (id: CE0170) is ALGPAATEE (204–210, 212, 213) TA (216–217); parameters of Pep-3D-Search are similarity mode and AHA (distance threshold = 4).

**No.**	**Residues and Locations of Candidate**												**Score**
1*	D197	I201	**L205**	**G206**	**P207**	**A209**	**E213**	**T216**	M215	R162	D163	D166	2.1385
2*	D197	I201	**L205**	**G206**	**P207**	**A209**	**E213**	**T210**	L211	Y169	R167	D166	2.1385
3*	D197	I201	**L205**	**G206**	**P207**	**A209**	**E213**	**T216**	**E212**	M215	R162	D163	2.1385
4*	D197	I201	K203	**G206**	**P207**	**A209**	**E213**	**T216**	**E212**	L211	Y169	D166	2.1385
5*	D197	I201	K203	**G206**	**P207**	**A209**	**E213**	**T210**	M214	M215	R162	D166	2.1385
6*	D197	I201	**L205**	**G206**	**P207**	**A208**	**A209**	**T210**	L211	Y169	V165	D163	2.1385
7*	D197	I201	**L205**	**G206**	**P207**	**A208**	**A209**	**T210**	**E213**	L211	Y169	D166	2.1385
8*	D197	I201	**L205**	**G206**	**P207**	**A209**	**E213**	**T210**	M214	**A217**	**T216**	**E212**	2.1385
9*	D197	I201	**L205**	**G206**	**P207**	**A209**	**E213**	**T216**	**A217**	Q219	M215	**E212**	2.1385
10*	D197	I201	**L205**	**G206**	**P207**	**A209**	**E213**	**T210**	**E212**	M214	V191	E187	2.1385

### The searching capability of Pep-3D-Search

**Figure 4:**
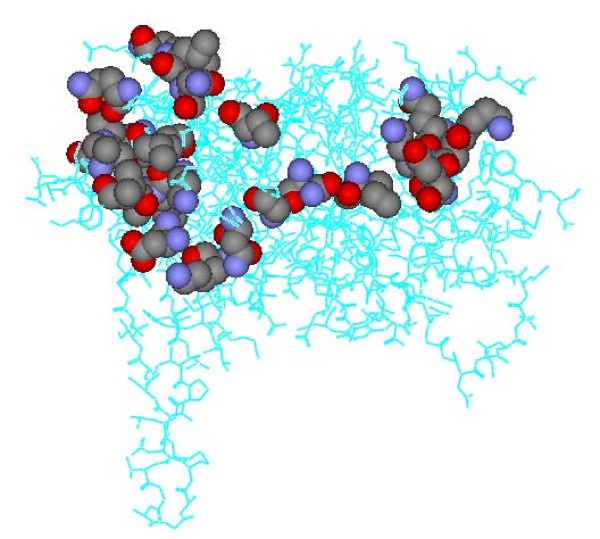
**A path on the gp120 (the envelope protein of HIV) surface**. The path on the gp120 surface, which is used to evaluate the searching capability of Pep-3D-Search, is composed of 25 residues, E351 S347 K343 Q344 K348 I272 N234 G237 N94 K97 D99 M100 K487 V489 L226 V488 A224 A219 Y217 C218 Q246 V84 L86 N88 T240, in which the Euclidian distance of the any two neighbor residues is less than or equal to 7.5 Å.

In general, the searching algorithm has a great impact on the effectiveness and efficiency of an epitope prediction program. Therefore it is the most important part of the whole design process. In Pep-3D-Search, the ACO algorithm, a kind of heuristic algorithm, is employed for searching mimotopes or motifs on an antigen surface. In order to evaluate the capability of the ACO algorithm for searching the target paths with various lengths on the antigen surface, we took gp120 (the envelope protein of HIV; chain G; PDB id: 1g9m; the residue number of the antigen is 304, see Table 2) as the target antigen and randomly selected the paths with lengths from 9 to 25 (odd numbers) residues on the antigen surface as the search goals. As shown in Figure 4, a path on the gp120 surface with 25 residues is localized firstly, E351 S347 K343 Q344 K348 I272 N234 G237 N94 K97 D99 M100 K487 V489 L226 V488 A224 A219 Y217 C218 Q246 V84 L86 N88 T240, in which the Euclidian distance of any two neighbor residues is less than or equal to 7.5 Å. From this path, 9 sub-paths with lengths from 9 to 25 (odd numbers) residues were randomly selected as the test cases (see Table 7 and Supplementary Table S6 in Additional file 1). Here, we describe one experiment in detail to explain the search process of the target path with 21 residues on the gp120 surface. The target path is E351 S347 K343 Q344 K348 I272 N234 G237 N94 K97 D99 M100 K487 V489 L226 V488 A224 A219 Y217 C218 Q246 (see Table 7). We used the target path itself and mutations of it as input sequence for Pep-3D-Search to localize the target path on the gp120 surface. Some residues on the original sequence were randomly changed (the mutation rates vary from 10% to 30%). From Table 7, it can be seen that Pep-3D-Search quickly localized the target path with 5000 iteration numbers. When the input sequence was the target path itself (ESKQKINGNKDMKVLVAAYCQ), the path localized by Pep-3D-Search with the iteration number of 5000 was **E351 S347 K343 Q344 K348 I272 N234 G237 N94 K97 D99 V488 K487 V489 L226 **V245 **A224 A219 Y217 **C247 **Q246**, which overlaps 19 of the 21 residues in the target path; when the iteration number was set to 25000, Pep-3D-Search precisely localized the target path. When the iteration number was 30000, the path localized by Pep-3D-Search was **E351 S347 K343 Q344 K348 I272 N234 G237 N94 K97 D99 M100 K487 V489 L226 V488 A224 A219 Y217 **C247 **Q246**. Though the twentieth residue (C247) on the localized path is not identical with the corresponding one (C218) on the target path in that position, they are all Cysteine. When a mutated sequence is used as input sequence, Pep-3D-Search still localized the region of the target path. For example, using ESK**DR**INGN**C**DMKV**H**VAAY**A**Q (the mutation rate is 25%) as input, Pep-3D-Search gave the top-ranked output: E267 T232 K231 N229 K485 F233 **N234 G237 N94 **___ **D99 M100 K487 V488 **___ I491 G222 **A219 **F223 **A224 Q246 **with 10000 iteration numbers. As shown in Table 7, although Pep-3D-Search got the worst result in the test case, it overlaps 10 of 21 residues in the target path.

**Table 7:** Evaluation of the Pep-3D-Search's searching capability.

**Mutation**	**Input sequence**	**TP/PE**					
		**IT = 5000**	**IT = 10000**	**IT = 15000**	**IT = 20000**	**IT = 25000**	**IT = 30000**
No	ESKQKINGNKDMKVLVAAYCQ	19/21	19/21	19/21	19/21	21/21	20/21
10%	ESKQ**R**INGNKDMKVL**P**AAYCQ	19/21	15/21	19/21	18/21	15/21	19/21
15%	ES**N**QKINGNK**S**MKVLVAA**M**CQ	16/21	20/21	18/21	16/21	16/21	17/21
20%	E**N**KQKI**D**GNKD**C**KVLV**P**AYCQ	18/21	15/21	15/21	15/21	18/21	15/21
25%	ESK**DR**INGN**C**DMKV**H**VAAY**A**Q	17/21	10/21	15/21	10/21	10/21	10/21
30%	**A**SKQK**LR**GNK**N**MKVL**C**A**C**YCQ	14/21	12/21	14/21	15/21	15/21	14/21

In the test case, the target path of 21 residues in length on the surface of the protein 1g9m (chain G) is E351 S347 K343 Q344 K348 I272 N234 G237 N94 K97 D99 M100 K487 V489 L226 V488 A224 A219 Y217 C218 Q246.

TP: number of true positives; PE: number of residues in the predicted epitope; IT: iteration number of Pep-3D-Search.

The experiments of other eight test cases for assessing Pep-3D-Search's searching capability are all based on similar procedures to the one described above. Those experimental results are listed in Supplementary Table S6 [see Additional file 1]. The experiments demonstrate the excellent search capability of Pep-3D-Search, especially when the length of the query sequence becomes longer; the iteration numbers of Pep-3D-Search for localizing the target paths on the protein surface did not change significantly. Thus, Pep-3D-Search can be used for quickly localizing the epitope regions mimicked by longer mimotopes (more than 20-residues), and the proposed ACO algorithm has further potential in other applications involving sequence-structure alignment.

## Discussion

In this study we developed a method, Pep-3D-Search, for epitope prediction based on mimotope and motif analysis. An ACO algorithm was proposed for aligning a 1D mimotope sequence (or a motif sequence) to the 3D structure of an antigen, and P-value calculation based screening strategy and DFS algorithm based clustering strategy were employed in localizing epitope candidate regions. Compared with competing methods, our Pep-3D-Search adopts a simple and natural strategy to deal with matches, gaps and deletions in aligning a sequence to an antigen surface, which makes it more efficient and effective, not only for sequence search, but also for motif discovery.

We conducted different sets of experiments to assess our method's performance. The results show that our method is comparable to other similar methods. In some test cases, our method is superior to the others or can provide complementary information to them. On the other hand, in order to examine the searching capability of our method, a set of test cases with different-length sequences was constructed. The experiment showed that our method has excellent capability in searching sequences on a structure, especially when the length of the query sequence becomes longer (up to 25 residues); the iteration numbers of Pep-3D-Search for precisely localizing sequence did not change significantly. Thus the method has further potential for localizing the epitope regions mimicked by longer mimotopes. For example, using an mRNA display technique, one can obtain affinity-selected peptides of more than 20 residues against an antibody [50]. Moreover, the method also has potential for other applications, such as querying pathways in protein-protein interaction networks [51]. The Pep-3D-Search algorithm depends on several parameters that may influence its prediction accuracy, such as iteration number, gap penalty and distance threshold defining two neighbor residues. However, because of the limited availability benchmark datasets, we only examined a limited set of values for each parameter and were constrained in properly learning these parameters. In our experiments, varying these parameters within a reasonable range did not significantly influence the prediction results (see Table 3 to 5).

The Pep-3D-Search algorithm is basically divided into three steps: generating random paths on the surface graph of an antigen for P-value calculation (which is not needed for motif analysis), searching the optimal paths for each mimotope (or a motif), and clustering these paths into several epitope candidates. The running time of the algorithm mainly depends on the number of graph edges, the number of mimotopes, the length of each mimotope (or the motif), and the number of generated random paths for P-value calculation. For a mimotope with 14 or 15 amino acids, generating 10^6 ^random paths to obtain the empirical distribution of alignment scores for P-value calculation may take about 10 minutes (using a PC with a Intel Core 2 processor at 1.86 GHz); searching the optimal paths may take few minutes (the iteration number is 20000 in default); clustering paths can complete in a few seconds. So the main computational burden of the algorithm comes from the P-value calculation.

Theoretically, the estimation of the statistical parameters for an alignment score distribution function requires a large number of random paths on the surface graph of the antigen for aligning to the mimotopes. Actually, the number of the paths generated at random is determined according to a given time limit, so that the algorithm can make a trade-off between computational time consumed and the accuracy of the final results. We set the number to 10^6 ^in default. In general, when a set of mimotopes is to be analyzed, the running time of the algorithm will linearly increase with the number of mimotopes. However, because a collection of paths generated at random for P-value calculation can be used by all those mimotopes in the same length in the set of the mimotopes, the actual running time of the algorithm is much shorter in practice. 

We plan to improve our method by further research in at least four areas: 1) by improving the method to identify surface-exposed residues in an antigen; 2) by attempting more effective strategies for searching a path and dealing with matches, gaps and deletions in aligning a sequence to antigen surface in the ACO algorithm; 3) by choosing a better amino-acid substitution matrix in scoring procedure for a specialized application; and 4) by studying more efficient methods for P-value calculation.

## Conclusion

This research makes two valuable contributions to the field of epitope prediction. Firstly, a promising ACO algorithm was proposed to align a sequence or a motif to an antigen surface. Secondly, an application program, Pep-3D-Search, was developed for epitope prediction based on mimotope or motif analysis. As a stand-alone program in this area, Pep-3D-Search is publicly accessible [see Additional file 2]. The program was tested and evaluated by several datasets [see Additional file 1, 3, 4 and 5]. The results indicate that Pep-3D-Search is comparable to other similar tools.

## Availability and requirements

Project name: Pep-3D-Search

Project's homepage: 

Operating system: Windows XP Professional with Service Pack 2(or later) with Microsoft .NET Framework 1.1(or later) installed

Programming language: Visual Basic.Net

License: GNU GPL

Any restrictions to use by non-academics: license needed for commercial use

## Authors' contributions

YXH designed the algorithm, performed the experiments and the analysis, and drafted the manuscript. YLB conceived of this study and discussed and suggested for algorithm improvement. SYG and YW collected the test data and carried out part of the experimental work and participated in writing the manuscript. CGZ designed research and contributed ideas. YXL supervised and directed the development process of the whole project and revised the manuscript critically. All authors have read and approved the final manuscript.

## Supplementary Material

Additional file 1 **Supplementary experiment-results.** The file contains supplementary tables S1 to S6. Click here for file

Additional file 2 **Source code, test datasets, Pep-3D-Search toolkit and operation manual.** The file is a ZIP archive containing the Visual Basic source code for Pep-3D-Search, licensed under the GNU General Public License. It also contains the test datasets, the Pep-3D-Search toolkit and the operation manual (in PDF format) of Pep-3D-Search. Updated versions will be available at . Click here for file

Additional file 3 **An example of predicting epitopes based on mimotope analysis.** The file is a ZIP archive containing all materials to predict the epitopes in the test case 1n8z using Pep-3D-Search based on mimotope analysis. Click here for file

Additional file 4 **An example of predicting epitopes based on motif analysis.** The file is a ZIP archive containing all materials to predict the epitopes in the test case 1e6j using Pep-3D-Search based on motif analysis. Click here for file

Additional file 5 **An example of evaluating the searching capability of Pep-3D-Search.** The file is a ZIP archive containing all materials to evaluate the Pep-3D-Search's searching capability by localizing the target path of 21 residues in length on the surface of the protein 1g9m (chain G) with original and mutated sequences of the target path as inputs. Click here for file
