# Supplementary material for: Pep-3D-Search: a method for B-cell epitope prediction based on mimotope analysis
Source: BMC Bioinformatics. 2008 Dec 16;9:538. doi: 10.1186/1471-2105-9-538 (PMC2639436; doi:10.1186/1471-2105-9-538)
Supplement: Additional file 1 — Supplementary experiment-results. The file contains supplementary tables S1 to S6. [file 1471-2105-9-538-S1.pdf]

Supplementary experiment-results

Contents

Table S1. Epitope prediction of the test case 1gc1 (chain G) based on motif mapping.

Table S2. Epitope prediction of the test case 1n8z (chain C) based on motif mapping.

Table S3. Epitope prediction of the test case 1iqd (chain C) based on motif mapping.

Table S4. Epitope prediction of the test case 1yy9 (chain A) based on motif mapping.

Table S5. Epitope prediction of the test case 2adf (chain A) based on motif mapping.

Table S6. The test cases and results for evaluating Pep-3D-Search's searching capability.

Table S1. Epitope prediction of the test case 1gc1 (chain G) based on motif mapping: motif sequence taken from Mimox is [LV]RP[LT][KR]LRE[LP][RT]X[-R]; native epitope recorded in CED (id: CE0058) is CK (119, 121) VTQAC (200, 202-205) RKQI (419, 421-423) KMYP (432, 434, 435, 437); parameters of Pep-3D-Search are similarity mode, CB (distance threshold =7).

No.	Residues and Locations of Candidate	Score
1*	L125 N197 P124 L122 **K432 I423 K421 Q422** I420 **R419** G329 A299	1.9795
2*	L116 K117 P118 V120 **K432 I423 K421 Q422** I420 **R419** G329 H330	1.9795
3*	L125 N197 P124 T123 **K121** L122 **K432** N425 I424 **K421** I420 **R419**	1.9795
4*	L125 N197 P124 L122 **K432 I423 K421 Q422 P437** A436 **C205** K117	1.9795
5*	L116 K117 P118 V120 **K121** L122 **K432** N425 I424 **K421 I423 Y435**	1.9795
6*	L125 N197 P124 L122 **K432** I424 **K421 Q422 P437** I439 S440 Q442	1.9795
7*	L125 N197 P124 L122 **K432 I423 K421 Q422** I424 N425 **G431 K121**	1.9795
8*	L125 N197 P124 L122 **K432 I423 Q422 K421** I420 **R419** C418 G329	1.9795
9*	L116 K117 P118 V120 **K432** L122 **K121** T123 P124 T198 C196 C126	1.9795
10*	L116 K117 P118 V120 **K432 I423 K421 Q422 P437** I439 P438 S440	1.9795

Table S2. Epitope prediction of the test case 1n8z (chain C) based on motif mapping: motif sequence taken from Mimox is [KV][DL]Y[HW][AY][DE]-GEIT; native epitope recorded in CED (id: CE0096) is PEADQ (557-561) DPPF (570-573) KFPDEEGACQP (593-603); Parameters of Pep-3D-Search are similarity mode, CA (distance threshold =7).

No.	Residues and Locations of Candidate	Score
1*	**K593 D570** Y568 H567 A566 E544 V575 K569 **G599 E598 A600 D596**	2.3587
2*	**K593 D570** Y568 H567 A576 E544 C565 K569 **G599 E597 D596 A600**	2.3587
3*	**K593 D570** Y568 H567 A566 Q546 P547 R530 G527 Q526 L525 V524	2.3587
4*	**K593 D570** Y568 H567 A566 Q546 C565 K569 **G599 E598 D596 Q602**	2.322
5*	**K593 D570** Y568 H567 A566 Q546 A564 C562 **A559** E544 V575 K569	2.322
6*	**K593 D570** Y568 H567 A576 R577 C578 **P595 G599** K569 V575 **A559**	2.322
7*	**K593 D570** Y568 H567 A566 Q546 Q548 N549 G550 S551 V563 A564	2.322
8*	V575 **D570** Y568 H567 A566 E544 C565 K569 **G599 E597 E598 D596**	2.2879
9*	V575 **D570** Y568 H567 A576 E544 C565 K569 **G599 E598 A600 P571**	2.2879
10*	K569 **D570** Y568 H567 A566 Q546 P547 R530 G527 R523 V524 V533	2.2879

Table S3. Epitope prediction of the test case 1iqd (chain C) based on motif mapping: motif sequence taken from Mimox is [NQKR][HST]RWSNRSS[ST]; native epitope recorded in CED (id: CE0176) is FTNMF (2196-2200) R2215 RPQV (2220-2223) SLLT (2250-2253) HQ (2315-2316); parameters of Pep-3D-Search are similarity mode, CB (distance threshold =7.5).

No.	Residues and Locations of Candidate	Score
1*	N2224 **Q2222 R2220** W2203 S2216 N2217 K2207 S2206 S2204 T2202	2.396
2*	N2224 **Q2316** R2220 W2203 S2216 N2217 K2207 S2204 S2206 T2191	2.396
3*	K2227 H2309 R2307 W2229 S2194 **Q2222 R2220** S2204 S2206 T2191	2.396
4*	K2227 H2309 R2307 W2229 E2228 N2224 **Q2222** S2194 S2193 T2191	2.396
5*	N2224 **Q2222 R2220** W2203 K2207 N2217 **R2215** S2216 **S2250 T2253**	2.379
6	Q2311 H2309 R2307 W2229 K2227 N2225 E2228 S2194 S2193 T2191	2.379
7*	K2207 S2204 **R2220** W2203 S2216 N2217 **R2215** A2201 **N2198** T2202	2.362
8*	**Q2222** Y2195 **R2220** W2203 S2216 N2217 K2207 S2206 A2188 T2191	2.361
9*	N2224 **Q2316 R2220** W2203 K2207 N2217 **R2215** A2201 S2216 **S2250**	2.346
10*	**N2198 F2196 R2220** W2203 K2207 N2217 **R2215** S2216 A2201 **T2197**	2.346

Table S4. Epitope prediction of the test case 1yy9 (chain A) based on motif mapping: motif sequence taken from Mimox: QW[DNQ][LR][SFW]SRX[LY]K; native epitope recorded in CED (id: CE0199) is R353 Q384 QHFVS (408-409, 412, 417-418) ISK (438, 440, 443) KIISN (465-468, 473); parameters of Pep-3D-Search are similarity mode, CA (distance threshold =7).

No.	Residues and Locations of Candidate	Score
1*	Q411 **F412 Q408 K407** F380 T406 R405 R403 I401 K430	2.2027
2	Q16 ___ D22 L25 F24 Q28 R29 N32 F54 K56	2.2027
3*	Q411 **F412** D436 **I438 S440 K465** K463 Q462 F457 K454	2.1777
4*	Q462 ___ D436 **F412** F380 T406 R405 R403 I401 K430	2.1397
5*	Q411 **F412** D436 **I438 S440 S468** N469 **I466 I467 K465**	2.1397
6*	**Q408 F412** D436 **I438 S440** G441 **K443** N469 **I466 K465**	2.0997
7	Q139 W140 R141 L149 F148 S150 N151 S92 Y93 N91	2.0717
8	Q139 W140 M152 M154 F156 D155 Q157 P130 L160 K105	2.0717
9*	**Q408 F412** D436 K463 Q462 D434 R403 S433 I401 K430	2.0697
10	D102 Y101 N129 L132 F156 D155 Q157 P130 L160 K105	2.0697

Table S5. Epitope prediction of the test case 2adf (chain A) based on motif mapping: motif sequence taken from Mimox is RT--[-FW]--[-LV]-SPWR; native epitope recorded in CED (id: CE0154) is ITTIDPWN (975-979, 981-983) DGFRY (1009, 1012, 1013, 1016, 1017) MH (1022, 1023); parameters of Pep-3D-Search are similarity mode, CA (distance threshold =7).

No.	Residues and Locations of Candidate	Score
1*	**R1016** T1019 S1020 **Y1017 F1013 D1009 T976 I975 T977** ___ **P981 W982** Q966	1.8459
2*	**R1016** T1019 S1020 **Y1017 F1013 T976 I975 T977 D979** ___ **P981 W982** Q966	1.8213
3*	**R1016** T1019 S1020 **Y1017 F1013 G1012 D1009 T976 T977** ___ **P981 W982 N983**	1.7644
4*	R1056 N1055 A1051 **G1012 F1013 D1009 T976 I975 T977** ___ **P981 W982** Q966	1.7342
5*	**R1016** T1019 S1020 **Y1017 F1013 T976 T977 I978 P981 N983** P986 ___ K988	1.7342
6*	**R1016** T1019 S1020 **H1023 Y1017 F1013 T976 I975 T977** ___ **P981 W982 N983**	1.7342
7*	R1053 A1049 A1048 **G1012 F1013 D1009 T976 I975 T977** ___ **P981 W982 N983**	1.7259
8*	**R1016** T1019 N1055 A1051 **G1012 F1013 T976 I975 T977** ___ **P981 W982** Q966	1.7096
9*	**R1016** T1019 S1020 E1021 P1027 R1026 Q966 I960 P986 **N983 P981 W982 H1023**	1.7096
10*	**R1016** T1019 S1020 **H1023 W982** Q966 R1026 A1029 K1031 S1030 P926 ___ R963	1.7096

Table S6. The test cases and results for evaluating Pep-3D-Search's searching capability.

MU	Input sequence	TP/PE					
		IT=5000	IT=10000	IT=15000	IT=20000	IT=25000	IT=30000
**Test case 1: the target path of 9 residues in length on the surface of the protein 1g9m (chain G), S347 K343 Q344 K348 I272 N234 G237 N94 K97**							
No	SKQKINGNK	9/9	9/9	9/9	9/9	9/9	9/9
10%	SKQK**F**NGNK	9/9**	9/9**	9/9**	9/9**	9/9**	9/9**
15%	SK**K**KING**D**K	9/9	6/9	9/9	9/9	9/9	6/9
20%	SKQ**QF**NGNK	4/9**	4/9**	4/9**	4/9**	4/9**	4/9**
25%	SK**RE**IN**D**NK	6/9*	6/9*	6/9*	6/9*	6/9*	6/9*
30%	**T**KQKI**H**GN**R**	8/9	8/9	8/9	8/9	8/9	8/9
**Test case 2: the target path of 11 residues in length on the surface of the protein 1g9m (chain G), D99 M100 K487 V489 L226 V488 A224 A219 Y217 C218 Q246**							
No	DMKVLVAAYCQ	11/11	11/11	11/11	11/11	11/11	10/11
10%	DMKVLVAA**H**CQ	11/11	10/11	10/11	10/11	10/11	11/11

15%	DMK**M**LVA**S**YCQ	10/11	10/11	10/11	10/11	11/11	11/11
20%	DMKVL**LC**AYCQ	9/11	9/11	9/11	9/11	9/11	9/11
25%	D**I**KV**L**VAAYC**K**	8/11	9/11	10/11	9/11	9/11	10/11
30%	DM**Q**VLVAA**WA**Q	9/11	9/11	9/11	9/11	9/11	9/11

Test case 3: the target path of 13 residues in length on the surface of the protein 1g9m (chain G), K348 I272 N234 G237 N94 K97 D99 M100 K487 V489 L226 V488 A224

No	KINGNKDMKVLVA	13/13	13/13	13/13	13/13	12/13	12/13
10%	KINGNK**S**MKVLVA	12/13	11/13	11/13	11/13	12/13	12/13
15%	KIN**N**NKDMKV**F**VA	12/13	12/13	11/13	12/13	12/13	11/13
20%	K**L**NGN**N**DMKVL**L**A	12/13	11/13	11/13	12/13	12/13	12/13
25%	KIN**ST**KDM**Q**VLVA	11/13	12/13	11/13	11/13	12/13	12/13
30%	**R**INGN**Q**DMK**L**LV**S**	12/13	12/13	12/13	13/13	13/13	13/13

Test case 4: the target path of 15 residues in length on the surface of the protein 1g9m (chain G), D99 M100 K487 V489 L226 V488 A224 A219 Y217 C218 Q246 V84 L86 N88 T240

No	DMKVLVAAYCQVLNT	13/15	13/15	14/15	14/15	13/15	14/15
10%	DMKVL**L**AAYCQVLNT	13/15	13/15	14/15	14/15	13/15	13/15
15%	DM**N**VLVAAYC**S**VLNT	11/15	10/15	14/15	11/15	11/15	11/15
20%	DMK**A**LVA**T**YCQVL**E**T	12/15	12/15	13/15	12/15	12/15	12/15
25%	D**I**KV**M**VAAY**T**QVLN**D**	9/15	9/15	11/15	9/15	9/15	9/15
30%	**N**MKVL**LP**AY**L**QV**I**NT	10/15	10/15	10/15	9/15	9/15	10/15

Test case 5: the target path of 17 residues in length on the surface of the protein 1g9m (chain G), E351 S347 K343 Q344 K348 I272 N234 G237 N94 K97 D99 M100 K487 V489 L226 V488 A224

No	ESKQKINGNKDMKVLVA	17/17	16/17	16/17	16/17	16/17	16/17
10%	E**L**KQKINGNK**N**MKVLVA	12/17	15/17	14/17	13/17	14/17	14/17
15%	ESKQ**I**ING**H**KDMKV**W**VA	13/17	15/17	15/17	16/17	15/17	16/17
20%	**R**SKQ**A**INGNK**Q**MKVL**Q**A	12/17	12/17	13/17	12/17	13/17	13/17
25%	ES**RS**KIN**N**NKD**L**KVLV**G**	11/17	9/17	12/17	11/17	12/17	13/17
30%	ESK**RQM**NGNKD**FS**VLVA	12/17	12/17	11/17	17/17	12/17	12/17

Test case 6: the target path of 19 residues in length on the surface of the protein 1g9m (chain G), K343 Q344 K348 I272 N234 G237 N94 K97 D99 M100 K487 V489 L226 V488 A224 A219 Y217 C218 Q246

No	KQKINGNKDMKVLVAAYCQ	18/19	17/19	18/19	18/19	18/19	16/19
10%	KQK**L**NGNKDMKVLVA**H**YCQ	14/19	15/19	15/19	15/19	16/19	16/19
15%	K**K**KINGNKD**V**KVLVAA**P**CQ	15/19	12/19	15/19	17/19	15/19	17/19

20%	KQK**G**NG**D**KDM**R**VL**C**AAYCQ	12/19	16/19	17/19	14/25	15/19	17/19
25%	KQ**M**ING**DN**DMKV**I**VAAY**V**Q	15/19	15/19	15/19	13/19	16/19	18/19
30%	**R**QK**L**NGNK**N**MKV**C**VA**G**YC**R**	14/19	12/25	11/19	15/25	15/19	15/19

Test case 7: the target path of 23 residues in length on the surface of the protein 1g9m (chain G), S347 K343 Q344 K348 I272 N234 G237 N94 K97 D99 M100 K487 V489 L226 V488 A224 A219 Y217 C218 Q246 V84 L86 N88

No	SKQKINGNKDMKVLVAAYCQVLN	20/23	22/23	21/23	19/23	20/23	23/23
10%	SKQKING**A**KDMKVLVAA**V**CQVLN	17/23	22/23	21/23	21/23	17/23	21/23
15%	SK**D**KINGNKDM**S**VLVAAYCQ**L**LN	22/23	21/23	19/23	22/23	17/23	16/23
20%	SKQ**A**ING**R**KDMK**L**LVA**I**YC**D**VLN	20/23	22/23	20/23	18/23	21/23	18/23
25%	**A**KQKI**HS**NKD**Q**KVLVA**T**YCQV**F**N	16/23	18/23	18/23	18/23	19/23	15/23
30%	S**NE**KINGNK**S**MKVL**L**AA**WA**QVL**R**	16/23	16/23	18/23	17/23	17/23	15/23

Test case 8: the target path of 25 residues in length on the surface of the protein 1g9m (chain G), E351 S347 K343 Q344 K348 I272 N234 G237 N94 K97 D99 M100 K487 V489 L226 V488 A224 A219 Y217 C218 Q246 V84 L86 N88 T240

No	ESKQKINGNKDMKVLVAAYCQVLNT	23/25	24/25	24/25	24/25	23/25	24/25
10%	**N**SKQKINGNKDM**E**VLVAAYCQVL**D**T	21/25	18/25	18/25	20/25	19/25	20/25
15%	ES**R**QKING**G**KDMKVLV**S**AYC**K**VLNT	24/25	24/25	20/25	22/25	22/25	21/25
20%	ESKQ**EM**NGNKD**F**KVLVA**R**YCQV**Q**NT	23/25	23/25	22/25	23/25	22/25	23/25
25%	E**A**KQKINGN**ES**MKV**TL**AAYCQVLN**S**	17/25	20/25	22/25	24/25	23/25	23/25
30%	ESK**R**KIN**N**NKD**V**KV**M**VAA**I**CQ**A**LN**L**	15/25	19/25	16/25	15/25	17/25	18/25

MU: mutation; TP: number of true positives; PE: number of residues in the predicted epitope; IT: iteration number of Pep-3D-Search.

*For the test cases, the best prediction was found in the second-ranked candidate.

**For the test cases, the best prediction was found in the third-ranked candidate.
