# Supplementary material for: Pep-3D-Search: a method for B-cell epitope prediction based on mimotope analysis
Source: BMC Bioinformatics. 2008 Dec 16;9:538. doi: 10.1186/1471-2105-9-538 (PMC2639436; doi:10.1186/1471-2105-9-538)
Supplement: Additional file 2 — Source code, test datasets, Pep-3D-Search toolkit and operation manual. The file is a ZIP archive containing the Visual Basic source code for Pep-3D-Search, licensed under the GNU General Public License. It also contains the test datasets, the Pep-3D-Search toolkit and the operation manual (in PDF format) of Pep-3D-Search. Updated versions will be available at . [file 1471-2105-9-538-S2.zip › Additional_file_2/Pep-3D-Search-20081205/Operation_manual.pdf]

Operation Manual of Pep-3D-Search

Operation guide

Pep-3D-Search is a useful application tool for computationally predicting epitopes based on mimotopes which are extracted from a phage display library or other combinatorial random peptide library such as mRNA display library. The algorithm input includes a 3D structure of antigen (a protein data bank (PDB) file), a set of mimotopes or a motif which can be derived from the set of mimotopes by using multiple sequence alignment tools such as ClustalW [35] or directly using Mimox web service (http://web.kuicr.kyoto-u.ac.jp/~hjian/mimox/). Pep-3D-Search firstly identifies all exposed residues of the given antigen and creates a surface graph of the antigen. After that, it can be used in two modes. The first is the mimotope mode, which searches for matching paths on antigen surface with each of the query mimotopes. Each of the matched paths is scored, and as a result, some of them are picked out as the putative candidate epitopes. The second is the motif mode, which directly maps the motif on to antigen surface and takes the top scoring paths as epitope candidates.

Pep-3D-Search is composed of four functional parts: (1) extracting the structure of the study antigen (i.e. the part of "Extract the Ag structure" in main menu); (2) obtaining the exposed residues of the study antigen (i.e. the part of "Computing the RSA" in main menu); (3) predicting epitopes based mimotope analysis (i.e. the part of "Search mimotopes to the surface of Ag" in main menu); (4) predicting epitopes based motif analysis (i.e. the part of "Search motifs on the surface of Ag" in main menu). Below, operation steps in each functional part are described in detail.

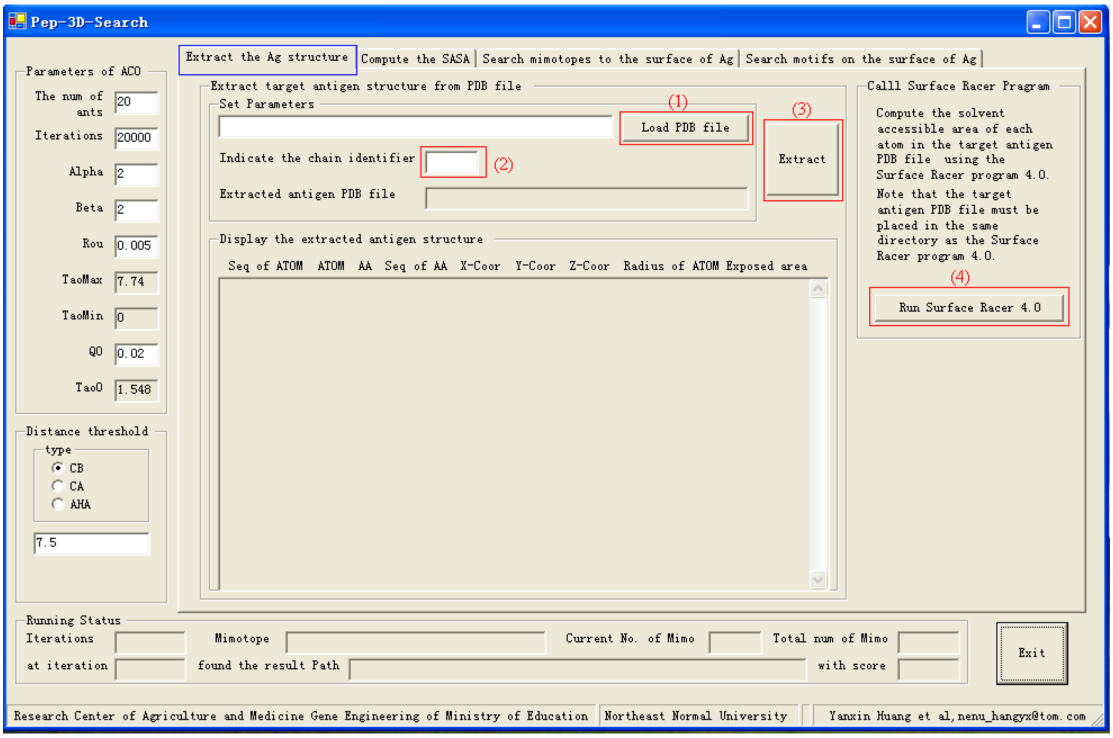

Figure 1: Extracting the study antigen structure from PDB file

1. Extracting the structure of the study antigen

The 3D structure of the study antigen is required by Pep-3D-Search. It can be derived from the structure files of complex of the antigen with other molecules (such as an antibody) if the structure of the complex is resolved and deposited in PDB database, or obtained by computational methods for protein structure prediction and modeling. Pep-3D-Search provides the function extracting a antigen structure from its complex file (PDB file). For example, when we intend to analyze the epitopes of envelope glycoprotein gp120 （chain G; PDB id: 1g9m）, we can obtain the 3D structure of envelope glycoprotein gp120 by Pep-3D-Search from the structure of HIV-1 HXBC2 gp120 envelope glycoprotein complexed with cd4 and induced neutralizing antibody 17b （PDB id: 1g9m）. Click on the button “Load PDB file” (the red box (1) in Figure 1); select 1g9m.pdb file, and then indicate the chain identifier G (the red box (2) in Figure 1); finally, Click on the button “Extract” (the red box (3) in Figure 1). Pep-3D-Search will extract the structure of chain G of 1g9m (i.e. the structure of envelope glycoprotein gp120) and save it in the directory same as the program Pep-3D-Search with name of PDB ID + chain identifier + “.pdb”. In this example, we will get the structure file of envelope glycoprotein gp120 “1g9m_g.pdb”. Note that, if the structure of the study antigen is obtained previously, this step can be skipped.

The next is to compute the solvent accessible area of each atom in the study antigen. This can be completed by clicking on the button “Run surface racer 4.0” (the red box (4) in Figure 1) or directly download Surface Racer program 4.0 from URL: http://www.pharmacy.umich.edu/tsodikovlab/index_files/Page756.htm. As a result, input the PDB file of the study antigen (e.g. 1g9m_g.pdb), and we will get the exposed residue file which name is the same as the PDB file but with the extension name ".txt" (e.g. 1g9m_g.txt).

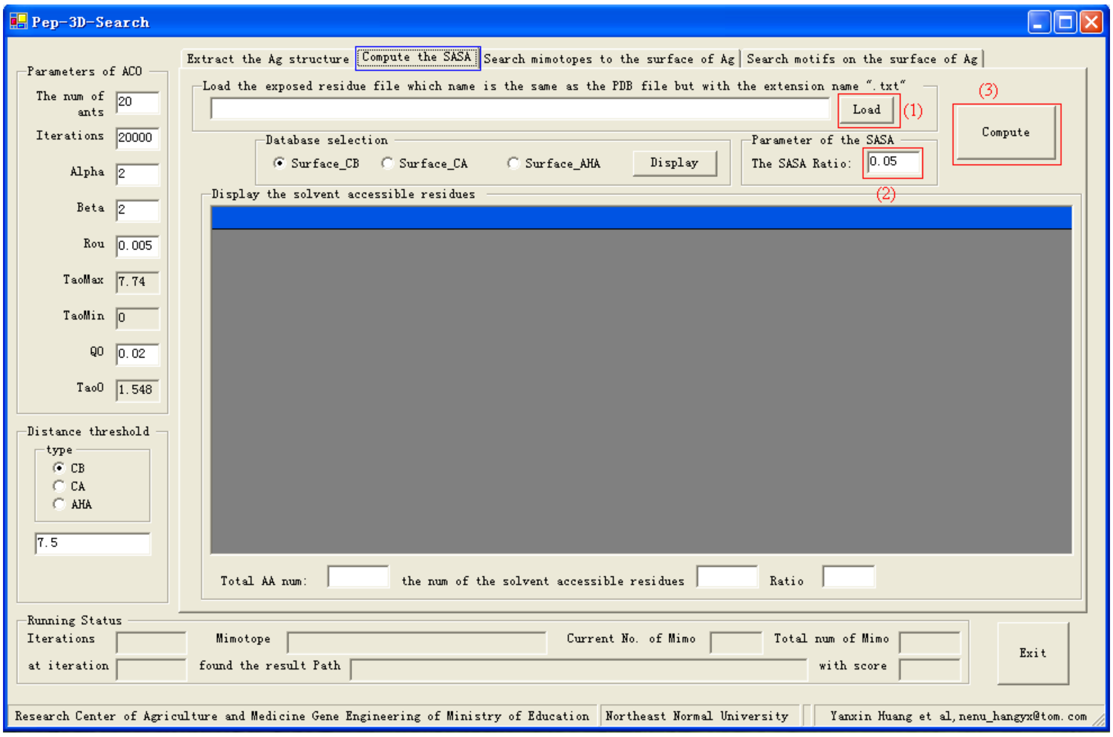

Figure 2：Load the result file of Surface Racer program 4.0 and the mimotope file

2、Compute solvent accessible residues

Click on the button "Load PDB file" (the red box (1) in Figure 2); select the exposed residue file which is gotten by Surface Racer program 4.0 (e.g. 1g9m_g.txt); check the threshold value which is used to determine whether or not a residue is the exposed (the red box (2) in Figure 2). If the solvent accessibility of a residue is greater than the threshold, then the residue is determined as exposed. In default, the threshold is set to 0.05. The greater the threshold is, the less the exposed residues are selected. Finally, click on the button "Compute" (the red box (2) in Figure 2) to complete the calculation of the exposed residues. For computation sake, the exposed residues are deposited in three different databases: Surface_CB, Surface_CA and Surface_AHA, and you can check the contents of them by clicking on button "Display" with proper radiobutton in checked status.

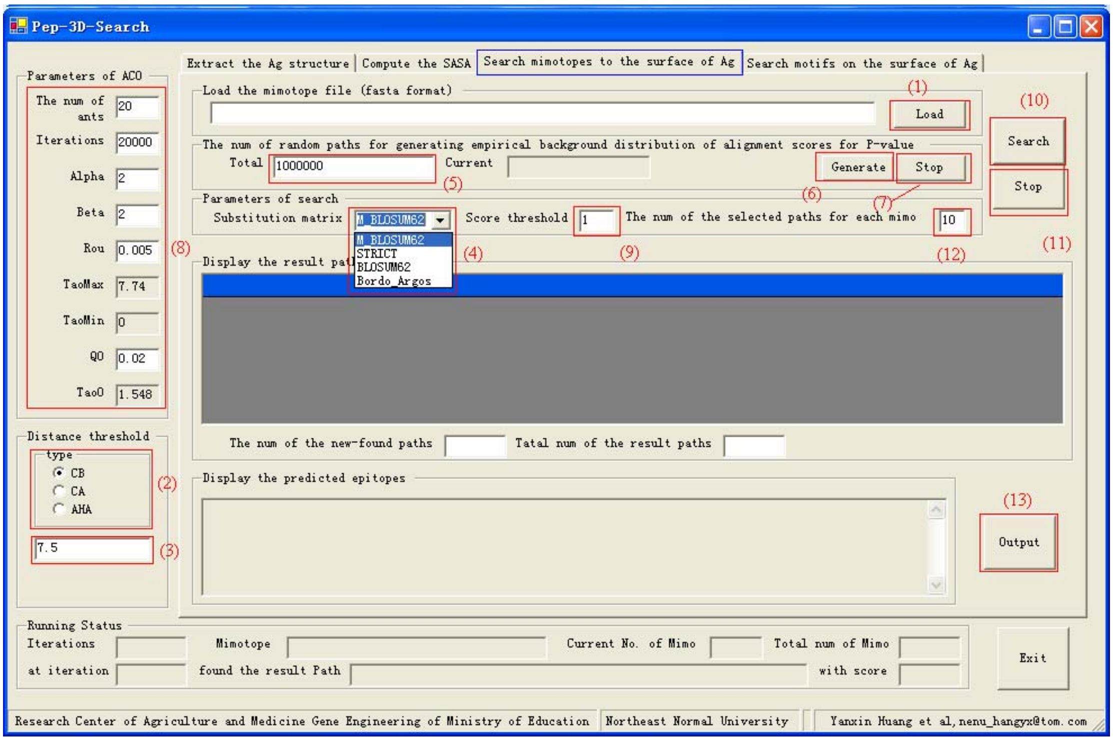

Figure 3：Predict epitopes based on mimotope analysis

3、Predict epitopes based on mimotope analysis

Epitope prediction based on mimotope analysis is basically divided into three stages: (1) generating random paths on the surface graph of antigen for P-value calculation; (2) searching the optimal paths for each mimotope; (3) outputting epitope candidates by clustering these paths. Below, the operation steps of the three stages are described.

3.1 Generate random paths on the surface graph of antigen for P-value calculation

Step 1: Click on the button "Load" (the red box (1) in Figure 3) to load the file of mimotopes. For example, when analyzing the epitopes of envelope glycoprotein gp120, we will select the file "17b_1G9M.txt" which contains all mimotopes obtained by biopanning phage-displayed random peptide library against the monoclonal antibody 17b.

Step 2: Select the method for calculating neighbour residue pairs on antigen surface (the red box (2) in Figure 3). The default is CB which takes the distance between the C_β atoms as the

distance between the two residues, which may better reflect the side chain position.

Step 3: Indicate the distance threshold of neighbour residue pairs on antigen surface (the red box (3) in Figure 3). For CB, the distance threshold is set to 7.5Å in default; for CA, the distance threshold is also set to 7.5Å in default; for AHA, the distance threshold is set to 4Å in default.

Step 4: Indicate the amino-acid substitution matrix (the red box (4) in Figure 3). The substitution matrix M_Blosum62 is used in default.

Step 5: Set the number of paths generated at random on the surface of antigen (the red box (5) in Figure 3). To obtain rational empirical distribution of alignment scores, the number should be as great as possible and it is set to 10^6 as default.

Step 6: Click on the button "Generate" (the red box (6) in Figure 3) to actually generate random paths on the surface of antigen and align them to the corresponding mimotopes. If you want to terminate the calculation, can click on the button "Stop" (the red box (7) in Figure 3)

Note that, the above stage may be a time-consuming procedure depending on the number of exposed residues, the number of mimotopes, the length of each mimotope, and the number of generated random paths.

3.2 Searching the optimal paths for each mimotope

Step 1: Under the condition of retaining the previously set parameters such as distance threshold invariable, indicate the parameters of the ant colony optimization (ACO) algorithm (the red box (8) in Figure 3).

(1) The number of ants. If the number of the exposed residues on the study antigen is greater than 400, the number of ants can be set to 30. Otherwise, the number of ants should be retained the default value 20.

(2) The iteration number of the algorithm. The greater the iteration number is, the better the result can be obtained. If the number of the exposed residues on the study antigen is less than 400, setting the iteration number to 20000 can generally be acceptable.

(3) Alpha (α). The parameters Alpha (α) and Beta(β) define the relative importance of the pheromone information and the heuristic information (default $\alpha=\beta=2$).

(4) Beta (β). The parameters Alpha (α) and Beta(β) define the relative importance of the pheromone information and the heuristic information (default $\alpha=\beta=2$).

(5) Rou (ρ). Rou (ρ) defines the speed of the pheromone on all edges decay after each iteration (default $\rho=0.05$).

(6) TaoMax ($\tau_{\max}$). The parameter value is calculated automatically based the used amino-acid substitution matrix.

(7) TaoMin ($\tau_{\min}$). The parameter value is calculated automatically based the used amino-acid substitution matrix.

(8) Q0. The parameter denotes the rate of ants choosing an edge with the biggest probability in each of their searching step. In general, Q0 is set in the range of 0 to 0.02 (default Q0=0.02).

(9) Tao0(τ_0).The parameter value is calculated automatically based TaoMax ($\tau_{\max}$) and TaoMin ($\tau_{\min}$).

Step 2: indicate the initial value of the score threshold (the red box (9) in Figure 3) which is used

to select the elite ants. The score threshold is adjusted automatically in running. However, you can indicate its initial value in this step and the initial value of the score threshold is set 1 in default.

Step 3: Click on the button “Search” (the red box (10) in Figure 3) to start the search procedure. If you want to terminate the procedure, can click on the button “Stop” (the red box (11) in Figure 3)

3.3 Output epitope candidates

Step 1: Indicate the number of the paths selected with each mimotope (the red box (12) in Figure 3). The number determines how many paths with respect to each mimotope is selected to create a set of the result paths and then to output epitope candidates by a clustering procedure.

Step 2: Click on the button “Output” (the red box (13) in Figure 3) to start the output procedure. The predicted result is not only displayed on the textbox of “Display the predicted epitopes”, but also written into a text file “Cluster.txt” in the directory same as the program Pep-3D-Search.

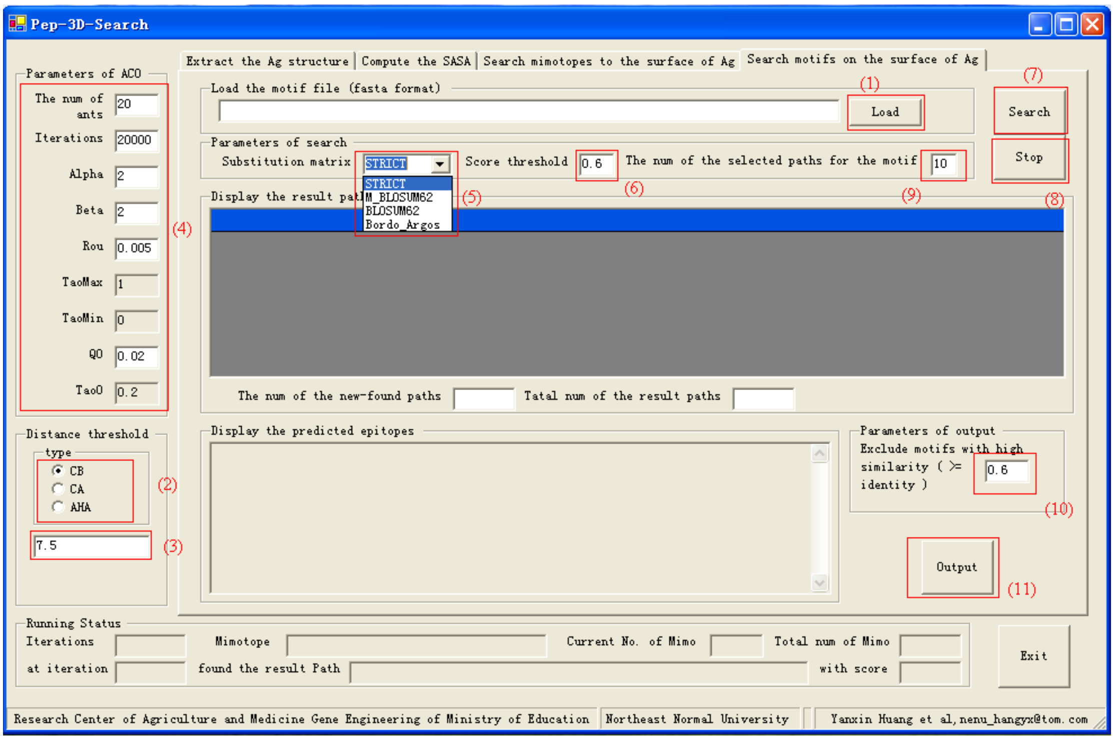

Figure 4：Output the predicted epitopes

4、Predict epitopes based on mimotope analysis

Epitope prediction based on motif analysis is basically divided into two stages: (1) searching the optimal paths for the motif; (3) outputting epitope candidates by screening these paths. Below, the operation steps of the two stages are described.

4.1 Searching the optimal paths for the motif

Step 1: Click on the button “Load” (the red box (1) in Figure 4) to load the file containing the motif. For example, when analyzing the epitopes of envelope glycoprotein gp120, we will

select the file "17b_1g9m_motif.txt" which contains the motif "CXX[LR][PR]TNX[TDE][TKR]L[RST]RC" derived from the set of mimotopes using Mimox web service (http://web.kuicr.kyoto-u.ac.jp/~hjian/mimox/).

Step 2: Select the method for calculating neighbour residue pairs on antigen surface (the red box (2) in Figure 4). The default is CB which takes the distance between the C_β atoms as the distance between the two residues, which may better reflect the side chain position.

Step 3: Indicate the distance threshold of neighbour residue pairs on antigen surface (the red box (3) in Figure 4). For CB, the distance threshold is set to 7.5Å in default; for CA, the distance threshold is also set to 7.5Å in default; for AHA, the distance threshold is set to 4Å in default.

Step 4: indicate the parameters of the ant colony optimization (ACO) algorithm (the red box (4) in Figure 4). The method of setting those parameters can refer to Step 1 in "3.2 Searching the optimal paths for each mimotope".

Step 5: Indicate the amino-acid substitution matrix (the red box (5) in Figure 4). In exact match mode, the substitution matrix STRICT is used in default; in similar match mode, the substitution matrix M_Blosum62 is used in default.

Step 6: indicate the initial value of the score threshold (the red box (6) in Figure 4) which is used to select the elite ants. The score threshold is adjusted automatically in running. However, you can indicate its initial value in this step and the initial value of the score threshold is set 0.6 in default.

Step 7: Click on the button "Search" (the red box (7) in Figure 4) to start the search procedure. If you want to terminate the procedure, can click on the button "Stop" (the red box (8) in Figure 4)

4.2 Output epitope candidates

Step 1: Indicate the number of the highest scoring paths (the red box (9) in Figure 4). The number determines how many highest scoring paths will be selected as epitope candidates.

Step 2: Indicate the threshold that is used to filter the set of epitope candidates (the red box (10) in Figure 4). In searching paths on the antigen surface, Pep-3D-Search will discover a great number of paths, whose scores are greater than the score threshold, and store those paths in the database. In order to exclude similar paths from the predicted results, Pep-3D-Search firstly selects the highest scoring path as an epitope candidate, and for each remaining paths, delete it if it is similar as the previously accepted paths (i.e. the number of its residues identical with the previously selected path is greater than the product of the threshold and the total residue number). In the same way, the next path is selected, …, until all epitope candidates are determined.

Step 3: Click on the button "Output" (the red box (11) in Figure 4) to start the output procedure. The predicted result is not only displayed on the textbox of "Display the predicted epitopes", but also written into a text file "Cluster.txt" in the directory same as the program Pep-3D-Search.

Availability and requirements

Project name: Pep-3D-Search
Project's homepage: http://kyc.nenu.edu.cn/Pep3DSearch/
Operating system: Windows XP Professional with Service Pack 2(or later) with Microsoft .NET Framework 1.1 (or Framework 2.0) installed
Framework 1.1 (dotnetfx.exe) can be download from:
http://www.microsoft.com/downloads/details.aspx?familyid=262D25E3-F589-4842-8157-034D1E7CF3A3&displaylang=en
Framework 2.0 (dotnetfx.exe) can be download from:
http://www.microsoft.com/downloads/details.aspx?FamilyID=0856EACB-4362-4B0D-8EDD-AAB15C5E04F5&displaylang=en)
Programming language: Visual Basic.Net
License: GNU GPL
Any restrictions to use by non-academics: license needed for commercial use
